# Improving Few- and Zero-Shot Reaction Template Prediction Using Modern Hopfield Networks

**DOI:** 10.1021/acs.jcim.1c01065

**Published:** 2022-01-15

**Authors:** Philipp Seidl, Philipp Renz, Natalia Dyubankova, Paulo Neves, Jonas Verhoeven, Jörg K. Wegner, Marwin Segler, Sepp Hochreiter, Günter Klambauer

**Affiliations:** † ELLIS Unit Linz, LIT AI Lab, Institute for Machine Learning, Johannes Kepler University Linz, Altenbergerstraße 69, Linz, Austria 4040; ‡ Janssen Pharmaceutica NV, High Dimensional Biology and Discovery Data Sciences, Janssen Research & Development, Turnhoutseweg 30, Beerse, Belgium 2340; ¶ Microsoft Research, 21 Station Road, Cambridge, United Kingdom CB1 2FB; § Institute of Advanced Research in Artificial Intelligence, Landstraßer Hauptstraße 5, Wien, Austria 1030; △ Janssen Research & Development, LLC, In-Silico Discovery and External Innovation (ISD&EI), 1 Cambridge Center, 255 Main St, Cambridge, Massachusetts 02142, United States

## Abstract

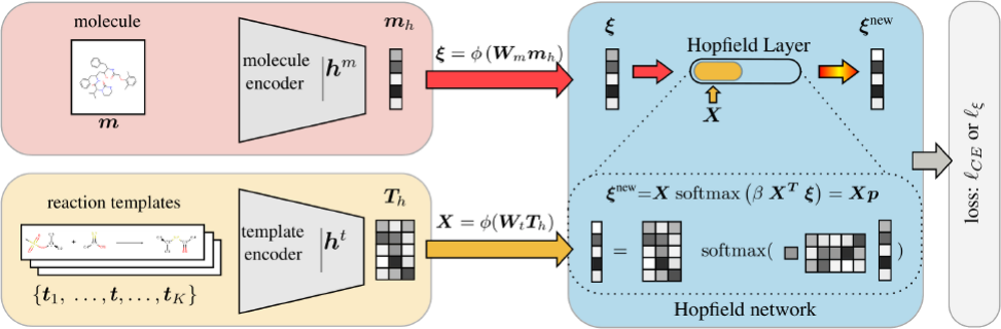

Finding synthesis routes for molecules of interest is essential in the discovery of new drugs and materials. To find such routes, computer-assisted synthesis planning (CASP) methods are employed, which rely on a single-step model of chemical reactivity. In this study, we introduce a template-based single-step retrosynthesis model based on Modern Hopfield Networks, which learn an encoding of both molecules and reaction templates in order to predict the relevance of templates for a given molecule. The template representation allows generalization across different reactions and significantly improves the performance of template relevance prediction, especially for templates with few or zero training examples. With inference speed up to orders of magnitude faster than baseline methods, we improve or match the state-of-the-art performance for top-*k* exact match accuracy for *k* ≥ 3 in the retrosynthesis benchmark USPTO-50k. Code to reproduce the results is available at github.com/ml-jku/mhn-react.

## Introduction

The design of a new molecule starts with an initial idea of a chemical structure with hypothesized desired properties.^1^ Desired properties might be the inhibition of a disease or a virus in drug discovery or thermal stability in material science.^2,3^ From the design idea of the molecule, a virtual molecule is constructed, the properties of which can then be predicted by means of computational methods.^4,5^ However, to test its properties and to finally make use of it, the molecule must be made physically available through chemical synthesis. Finding a synthesis route for a given molecule is a multistep process that is considered highly complex.^6,7^

To aid in finding synthesis routes, chemists have resorted to computer-assisted synthesis planning (CASP) methods.^6,8^ Chemical synthesis planning is often viewed in the retrosynthesis setting in which a molecule of interest is recursively decomposed into less complex molecules until only readily available precursor molecules remain.^9^ Such an approach relies on a single-step retrosynthesis model, which, given a product, tries to propose sets of reactants from which it can be synthesized. Early methods modeled chemical reactivity using rule-based expert systems.^8^ These methods, however, require extensive manual curation.^9−11^ Recently, there have been increased efforts to model chemical reactivity from reaction databases using machine learning methods.^9,12−15^

These efforts to model chemical reactions encompass a variety of different approaches. In one line of methods,^14,16−20^ the simplified molecular-input line-entry system (SMILES) representation^21^ of the reactants given that of the product is predicted, using architectures initially proposed for the translation between natural languages.^22,23^ Others exploit the graph structure of molecules and model the task using graph neural networks.^24,25^ A prominent line of work makes use of *reaction templates* which are graph transformation rules that encode connectivity changes between atoms during a chemical reaction.

In a template-based approach, reaction templates are first extracted from a reaction database or hand-coded by a chemist. If the product side of a template is a subgraph of a molecule, the template is called applicable to the molecule and can be used to transform it to a reactant set. However, even if a template can be applied to a molecule, the resulting reaction might not be viable in the laboratory.^11^ Hence, a core task, which we refer to as template-relevance prediction, in such an approach is to predict with which templates a molecule can be combined with to yield a viable reaction. In prior work, this problem has often been tackled using machine learning methods that are trained at this task on a set of recorded reactions.^9,11,26−32^

Template-based methods usually view the problem as a classification task in which the templates are modeled as distinct categories. However, this can be problematic as automatic template extraction leads to many templates that are represented by few training samples,^9,26^ Somnath et al.^25^ argued that template-based approaches suffer from bad performance, particularly for rare reaction templates. Segler^9^ and Struble et al.^10^ noted that machine learning (ML) has not been applied successfully for CASP in low-data regimes. To address the low-data issue, Fortunato et al.^26^ pretrained their template-relevance model to predict which templates are applicable and then fine-tuned it on recorded reactions in a database. This improved template-relevance prediction, especially for rare templates, as well as the average applicability of the top-ranked templates. Overall, a challenge of template-based methods arises from modeling reaction templates as distinct categories, which leads to many classes with few examples (see the section entitled “Methods”).

**Figure 1:**
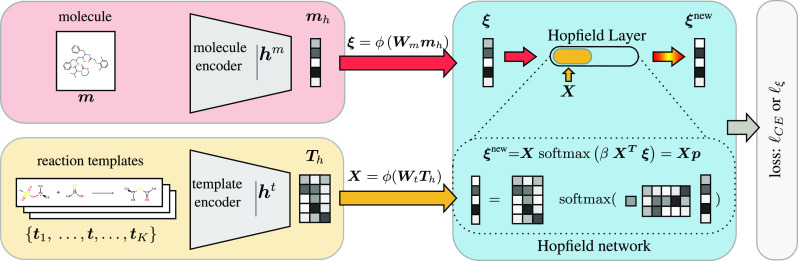
Simplified depiction of our approach. Standard approaches only encode the molecule and predict a fixed set of templates. In our modern Hopfield network (MHN)-based approach, the templates are also encoded and transformed to stored patterns via the template encoder. The Hopfield layer learns to associate the encoded input molecule, the state pattern **ξ**, with the memory of encoded templates, the stored patterns **X**. Multiple Hopfield layers can operate in parallel or can be stacked using different encoders.

To avoid the above-mentioned problems, we propose a new model that does not consider templates as distinct categories, but can leverage structural information about the template. This allows for generalization over templates and improves performance in the tasks defined in ref (26), especially for templates with few training samples and even for unseen templates. This model learns to associate relevant templates to product molecules using a modern Hopfield network (MHN).^33,34^ To this end, we adapted MHNs to associate objects of different modalities, namely input molecules and reaction templates. A depiction of our approach is illustrated in Figure 1.

In contrast to popular ML approaches, in which variable or input-dependent subsets of the data are associated,^22,33,35,36^ our architecture maintains a fixed set of representations, considered as a static memory independent of the input.

In this study, we propose a template-based method, which are often reported to be computationally expensive, because of the NP-complete subgraph-isomorphism calculations involved in template execution.^24−26,28^ To address this issue Fortunato et al.,^26^ Bjerrum et al.^28^ trained neural networks to predict which templates are applicable, given a molecule to filter inapplicable templates during inference. We find that using a substructure screen, i.e., a fast check of a necessary condition for a graph to be a subgraph of another improves inference speed, which may also be of interest for other template-based methods.

The main advance of our model over Fortunato et al.,^26^ Hasic and Ishida,^37^ or other template-based methods, is that by representing and encoding reaction templates we are able to predict relevant templates, even if few training data is available, which is a common issue in reaction datasets.

This work is structured as follows: In the “Methods” section, we propose a template relevance model that predicts template relevance by applying a multimodal learning approach using a modern Hopfield network. In the sections entitled “Template Prediction” and “Single-Step Retrosynthesis”, we demonstrate that our architecture improves predictive performance for template relevance prediction and single-step retrosynthesis. In the section enetitled “Inference Speed”, we show that our method is several times faster than baseline methods.

## Single-Step Retrosynthesis

The goal of *single-step retrosynthesis* is to predict sets of molecules that react to a given product.^7,38^ Since a molecule can be synthesized in various ways, this represents a one-to-many task. Performance in this setting is usually measured by *reactant top-k accuracy* using a reaction database. This metric measures the fraction of samples for which, given the product of a recorded reaction, the recorded reactants are among the top-*k* predictions. Given the one-to-many setting, small values of *k* might not be an optimal choice as there might exist scenarios where a good model receives low scores. Choosing a large *k* might result in a metric that is overly easy to optimize.

Template-based approaches predict reactant sets via reaction templates. A reaction template encodes atom connectivity changes during a chemical reaction and can be used to transform a product molecule to reactants, , where ***m*** is a product molecule, ***r*** represents a set of reactants and ***t*** a reaction template. The product side of a template encodes at which position in a molecule the template can be applied. A necessary condition for this is that the product side of the template is a substructure of the molecule of interest. If this is the case, a template is said to be *applicable* to the molecule. The product subgraph is then transformed according to the reactant side of the template and an atom-mapping between the two sides. Templates can be either hand-coded or automatically extracted from reaction databases, which yields an ordered set of *K* unique templates ***T*** = {***t***^*k*^}_*k*=1_^*K*^.

The aim of *template-relevance prediction* is to predict which templates result in a feasible reaction given a product. If this is the case, we say that a template is *relevant* to a molecule. While applicability is a necessary condition for relevance, it ignores the context of the whole molecule and thus substructures that might conflict with the encoded reaction (see Figure 1 in Segler and Waller^11^). In practice, applicability gives poor performance at relevance prediction (see Table 1, presented later in this work). To evaluate template-relevance predictions, we use *template top-k accuracy*, which given the product of a recorded reaction measures the fraction of samples for which the template extracted from the recorded reaction is among the top-*k* predicted ones.

Given relevance predictions for a product, reactant sets are obtained by executing top-scoring templates. We do not permit relevance prediction to rely on applicability calculations, because it is relatively slow to compute. Via this constraint, template top-*k* accuracy also incorporates information about the models ability to filter out nonapplicable templates. This information might be lost in reactant accuracy as template execution relies on a check for applicability. Other differences between the reactant/template accuracy can arise from multiple locations in which the correct template may be applied or incorrect templates leading to the correct reactants.

Multistep retrosynthesis can be achieved by applying single-step retrosynthesis recursively. One can decompose the desired molecule into less-complex molecules until only readily available precursor molecules remain.

## Methods

### Motivation

Many template-based methods^9,11,26−28^ consider a classification problem and predict templates using
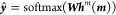
1where ***h***^*m*^(***m***) is a neural network (NN) that maps a molecule representation to a vector of size *d*_*m*_, which we call *molecule encoder*. ***W*** is a randomly initialized matrix, the last layer of the NN mapping from the molecule encoder to the predictive score of the template classes. Multiplication with  yields a score for each template ***t***_1_, ···, ***t***_*K*_. These scores are then normalized using the softmax function, which yields the vector . In this setting, different templates are viewed as distinct categories or classes, which makes the model ignorant of similarities between classes, which prevents generalization over templates. The high fraction of samples in reaction datasets that have a unique template can be problematic because they cannot contribute to performance. This problem might also appear for templates occurring only a few times, but to a lesser extent.

Instead of learning the rows of ***W*** independently, one could map each template to a vector of size *d*_*t*_ using a *template encoder*, ***h***^*t*^, and concatenate them row-wise to obtain . If *d*_*m*_ = *d*_*t*_, replacing ***W*** in the equation above yields
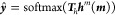
2which associates the molecule *m* with each template via the dot product of their representations. This allows generalization across templates, because the structure of the template is used to represent the class and the model can leverage similarities between templates. We adapt modern Hopfield networks^33^ to generalize this association of the two modalities, molecules and reaction templates.

### Modern Hopfield Networks

By going from eq 1 to eq 2, we have recast the problem of classifying a given molecule into a reaction template class into a content-based retrieval problem. Given a molecule ***m***, the correct address, or index, of the molecule’s associated template ***t*** in a database of templates ***T*** must be retrieved based on the chemical structure of the molecule. Such content-addressable, so-called associative memory systems realized as neural networks are called Hopfield networks.^39,40^ Their storage capacity can be considerably increased by polynomial terms in the energy function.^41−48^ In contrast to these binary memory networks, we use continuous associative memory networks with very high storage capacity. These modern Hopfield networks for deep learning architectures have an energy function with continuous states and can retrieve samples with only one update.^33,49^

For tackling retrieval problems, modern Hopfield networks perform several operations with so-called patterns, i.e., vector representations of the data points. A retrieval model based on a modern Hopfield network can be considered as a function *g* that returns the position ***ŷ*** of the retrieved pattern
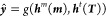
3The structure of the function *g* can be relatively complex,^33,34^ but consists of two main components: (a) a mapping to a *d*-dimensional associative space using linear embeddings ***W***_*m*_ and ***W***_*t*_ followed by a a non/linear activation ϕ. With these mappings, the state pattern **ξ** = *ϕ*(***W***_*m*_***h***^*m*^(***m***)) and stored patterns ***X*** = *ϕ*(***W***_*t*_***h***^*t*^(***T***)) are obtained. (b) An update function that performs the following operation,
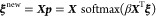
4where **ξ**^new^ is the retrieved pattern and the stochastic vector **p** associates the state pattern with the stored patterns and β > 0 is a scaling parameter (inverse temperature). The stored patterns ***X*** can be considered a memory of reaction templates. Other components, such multiple mappings to associative spaces in parallel, so-called heads, and iterative refinement of the retrieved patterns across multiple layers in the form of stacking are suggested by the powerful transformer architectures,^22^ which are also based on Hopfield networks.^33,34^ Multiple layers of parallel heads have been shown to be necessary for high predictive quality at natural language processing tasks.^22^ This design of the function *g* allows one to build a DL architecture that is potentially able to retrieve a correct reaction template from an arbitrary set of templates given a molecule.

All these above-mentioned operations and architectural components are implemented in the so-called “Hopfield layer”,^33^ whose design we use in our model and whose concrete settings are determined during hyperparameter selection. The matrices ***W***_*t*_ and ***W***_*m*_ that associate molecules and templates are learned during training of the model. In the following, we provide details on the architecture.

### Model Architecture

Our model architecture consists of three main parts: (a) a molecule encoder, (b) a reaction template encoder, and (c) one or more stacked or parallel Hopfield layers. First, we use a molecule encoder function that learns a relevant representation for the task at hand. For this, we use a fingerprint-based, e.g., extended connectivity fingerprint (ECFP),^50^ fully connected NN, ***h***_***w***_^*m*^(***m***) with weights ***w***. The molecule encoder maps a molecule to a representation ***m***_*h*_ = ***h***_***w***_^*m*^(***m***) of dimension *d*_*m*_.

Second, we use the reaction template encoder ***h***_**v**_^*t*^ with parameters ***v*** to learn relevant representations of templates. Here, we also use a fully connected NN with *template fingerprints* as input. These fingerprints are described in Section S3 in the Supporting Information. This function is applied to all templates ***T*** and the resulting vectors are concatenated column-wise into a matrix ***T***_*h*_ = ***h***_**v**_^*t*^(***T***) with shape (*d*_*t*_,*K*).

Finally, we use a single or several stacked or parallel Hopfield layers *g*(.,.) to associate a molecule with all templates in the memory. Hopfield layers consist of the option of layer normalization^51^ for **ξ** and ***X***, which is included as a hyperparameter. We also consider the scaling parameter β as a hyperparameter. The Hopfield layer then employs the update rule described by eq 4 through which the updated representation of the product molecule **ξ**^new^ and the vector of associations *p* is obtained. If multiple Hopfield layers are stacked, **ξ**^new^ enters the next Hopfield layer, for which additional template encoders supply the template representations. Parallel Hopfield layers use the same template encoder, but learn different projections ***W***_*t*_,***W***_*m*_, which is analogous to the heads in Transformer networks.

The simple model (eq 2) is a special case of our MHN and recovered if (a) ***W***_*t*_ and ***W***_*m*_ are the identity matrices and *d*_*t*_ = *d*_*m*_ = *d*, (b) the Hopfield network is constrained to a single update, (c) Hopfield networks are not stacked, i.e., there is only a single Hopfield layer, (d) the scaling parameter *β* = 1, (e) layer norm learns zero mean and unit variance and does not use its adaptive parameters, and (f) the activation function *ϕ* is the identity. The standard deep neural network (DNN) model (eq 1) is recovered if additionally the reaction templates are one-hot encoded, and the template encoder is linear.

In this study, we tested fingerprint-based fully connected networks for the molecule and template encoder. In principle, one could use any mapping from molecules/templates to vector-valued representations for these components, for example, raw fingerprints, graph neural networks^52^ or SMILES arbitrary target specification (SMARTS)-based RNNs,^53^ or Transformers.^22^

### Loss Function and Optimization

Given a training pair (***m***,***t***) and the set of all templates ***T***, the model should assign high probability to ***t***, based on ***m*** and ***T***. We encode this objective by the negative log-likelihood: −log *p*(***t***|***m***,***T***). The probability of each template in ***T*** is encoded by the corresponding element of the vector of associations ***p*** of the last Hopfield layer. In the simple case of a single correct template, this is equivalent to the cross-entropy loss _CE_(***y***,***p***) between the one-hot encoded label vector ***y*** and the predictions ***p***. In the case of multiple parallel Hopfield layers, we use average pooling across the vectors ***p*** supplied from each layer. We provide a general definition of the loss in terms of retrieved patterns and details in the section entitled “Related Work”.

The parameters of the model are adjusted on a training set using stochastic gradient descent on the loss with respect to ***W***_*t*_,***W***_*m*_,***w***,*v* via the AdamW optimizer.^54^ We train our model for a maximum of 100 epochs and then select the best model with respect to the minimum cross-entropy loss in the case of template-relevance prediction or maximum top-1 accuracy for single-step retrosynthesis on the validation set.

We use dropout regularization in the molecule encoder ***h***^*m*^ for the template encoder ***h***^*t*^, as well as for the representations in the Hopfield layers. We employ L2 regularization on the parameters. A detailed list of considered and selected hyperparameters is given in Tables S2 and S3 in the Supporting Information.

We added a computationally inexpensive fingerprint-based substructure screen as a post-processing step that can filter out a part of the nonapplicable templates. For each product and the product side of each template, we calculated a bit-vector using the “PatternFingerprint” function from RDKit.^55^ Each bit set in this vector specifies the presence of a substructure. For a template to be applicable, every bit set in the template fingerprint also must be set in the product fingerprint, which is a necessary condition for subgraphs to match. We chose a fingerprint size of 4096, as we did not observe significant performance gains for larger sizes, as can be seen in Figure S2 in the Supporting Information.

### Related Work

From the perspective of attention-based machine learning,^22^ our model can be seen as an attention mechanism that learns to attend elements or patterns of an external memory. The model proposed in Dai et al.^29^ is similar to our approach, because it also makes use of the templates’ structures and could even be seen as a special case, in which the memory of reaction templates is assembled based on logical operators. There are further restrictions on the structures of the encoder networks, which our work demonstrates are unnecessary. Because of the fact that representations of data from two different modalities, reactions and molecules, are learned, our approach also resembles the contrastive learning approaches taken in CLIP^36^ and ConVIRT,^56^ in which associated pairs of images and texts are contrasted against nonassociated pairs. Our adaption of MHN to maintain a static memory complements previous contrastive learning^35,57^ approaches using a memory.^58−61^ To embed molecule and reaction representations in the same latent space by maximizing the cosine similarity of reactions relevant to a given molecule has also been suggested by Segler.^9^ We also considered a contrastive learning setting using the InfoNCE loss;^35^ however, this led to slightly inferior results (see the Supporting Information).

### Data and Preprocessing

All datasets used in this study are derived from the United States Patent and Trademark Office (USPTO) dataset, extracted from the U.S. patent literature between 1976 and September 2016 by Lowe.^62^ This dataset contains 1.8 million text-mined reaction equations in SMILES notation^21^ and consists of reactions recorded in the years from 1976 to 2016. Reaction conditions and process actions are not included. For evaluating template relevance prediction, we use the preprocessing procedure described in ref (26). Templates are extracted using rdchiral.^63^ This results in two datasets: *USPTO-sm*, which is based on USPTO-50k,^64^ and *USPTO-lg*, which is based on *USPTO-410k*.^65^*USPTO-50k* contains only the 50 most populated reaction types, yielding a much simpler dataset than *USPTO-lg*, which is more diverse and entails multireactant reactions, which leads to many different templates

**Figure 2:**
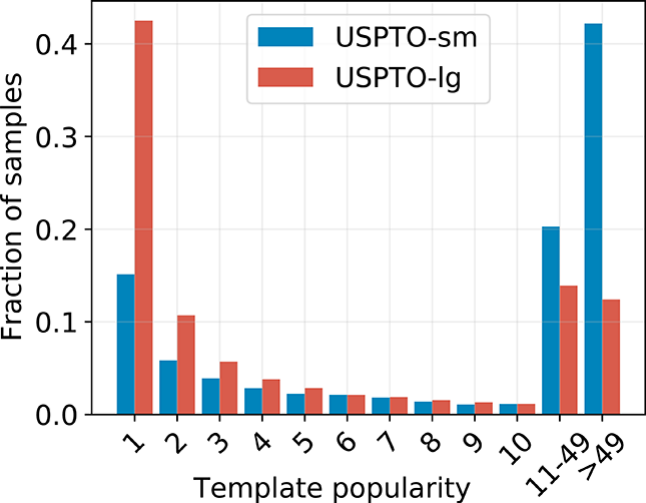
Histogram showing the fraction of samples for different template frequencies. The leftmost red bar indicates that over 40% of chemical reactions of *USPTO-lg* have a unique reaction template. The majority of reaction templates are rare.

For evaluating single-step retrosynthesis, we use *USPTO-50k* as preprocessed in ref (31). For this set, we also extract templates using rdchiral,^63^ but only for the train and validation split, to prevent test data leakage. Figure 2 displays the fraction of samples for different template frequencies for *USPTO-sm/USPTO-lg*. A detailed description of the datasets and their preprocessing can be found in Section S3 in the Supporting Information.

## Experiments and Results

### Template Prediction

In this section, we evaluate different models in the setup by Fortunato et al.^26^ Here, the aim is to predict the correct reaction templates, with template top-*k* accuracy used as a metric. In contrast to reactant prediction, this allows a more fine-grained analysis of the template ranking obtained by the models, because it ignores errors stemming from multiple potential application locations. The evaluation of our model in the full reactant prediction task is delayed to the next section. In their study, Fortunato et al.^26^ mainly compared two models. First, a fully connected network with a softmax output in which each output unit corresponds to a reaction template, conceptually similar to the model introduced in ref (11). We refer to this model as DNN. The second method is identical to the above, but instead of randomly initializing the weights, pretraining on a template applicability task (DNN+pretrain) is done. We extend this model by the addition of a template encoder and the MHN to associate the entities. We refer to models of the latter type as MHN while calling the former DNN. We also introduced the fingerprint filter (FPF) as a post-processing step. The choice of (a) model type, (b) the use of the FPF, and (c) the pretraining results in 2^3^ = 8 model variants. For all model variants, hyperparameters were adjusted on the validation set, as described in Section S3. We start with a general performance analysis and then investigate how rare templates affect the performance.

#### Overall Performance

The upper section of Table 1 shows the performance of these eight variants on *USPTO-sm* and *USPTO-lg*. Overall, it can be seen that the use of MHN and FPF yields large performance improvements over the methods evaluated in ref (26). Most notably, the top-100 accuracy increases by 10% on *USTPO-sm* and 18% on *USPTO-lg*. The plain MHN model without both FPF or pretraining has higher top-*k* accuracy for most values of *k* and both datasets, except for top-1 accuracy on *USPTO-lg*, showing the isolated performance gains by the model type. We will further investigate where these performance differences stem from below. Furthermore, the FPF yields non-negligible accuracy improvements for all models. Interestingly, pretraining and the FPF seem to complement each other in predicting applicability for the DNN models, rather than one of them being redundant. While pretraining yields non-negligible performance increases for the DNN models, the effect on the MHN model performance seems rather limited.

**Table 1:**
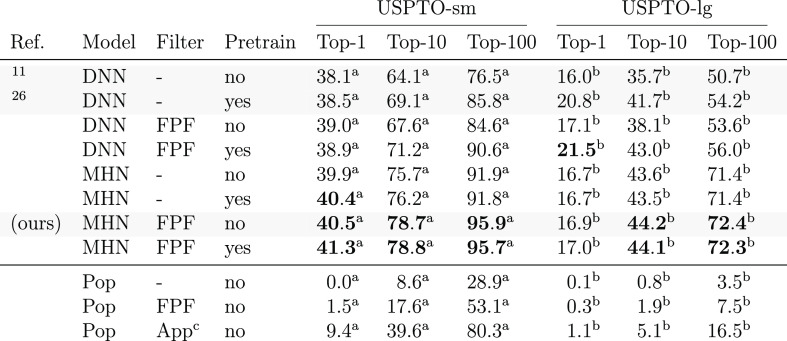
Template Top-*k* Accuracy (%) of Different Method Variants on *USPTO-sm* and USPTO-lg*

* “Model” indicates how the templates were ranked. “Filter” specifies if and how templates were excluded from the ranking via FPF or an applicability check (App). Pre-train indicates whether a model was pre-trained on the applicability task. Error bars represent confidence intervals on binomial proportions. The gray rows indicate methods specifically proposed here or in prior work.

a Width of 95% confidence interval <1.3%.

b Width of 95% confidence interval <0.4%.

c Note that the applicability filter violates modeling constraints from the section entitled “Single-Step Retrosynthesis”.

In the lower part of Table 1, we show the performance of a simple popularity baseline. This baseline predicts templates based on their occurrence in the training set. The last row shows that a plain applicability check is not sufficient for high performance. We include additional results in Section S3.

#### Rare Templates: Few- and Zero-Shot Learning

**Figure 3:**
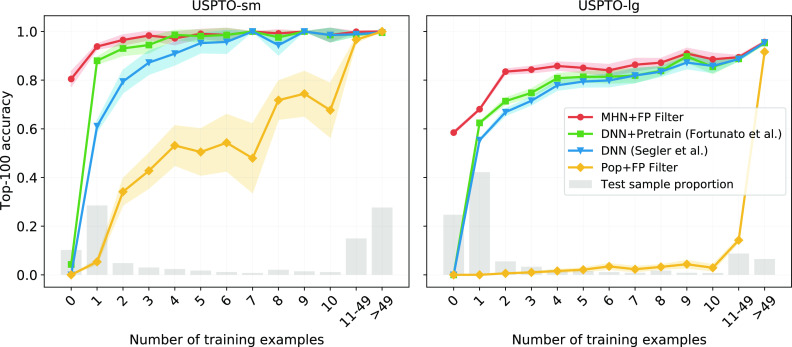
Top-100 accuracy for different template popularity on the *USPTO-sm*/*USPTO-lg* datasets. The gray bars represent the proportion of samples in the test set. Error bars represent 95% confidence intervals on binomial proportion. Our method performs especially well on samples with reaction templates with few training examples.

Given the propensity of rare template samples in the used datasets, we next show how the predictive performance varies with template popularity. Figure 3 shows the top-100 accuracy for different subsets of the test set, which were grouped according to the number of training samples with the same template. For improved clarity, we only include four of the above methods: DNN, DNN+pretrain, MHN+FPF, and the popularity baseline. Figure S1 in the Supporting Information shows all eight model variants. Especially for samples with rare templates, the performance gap between our method and the compared ones is large. As expected, in the experiments on template relevance prediction (see the section entitled “Template Prediction” and Figure 3), the DNN models and the popularity baseline perform poorly for samples with templates not seen during training. The MHN model, on the other hand, achieves far above random accuracy on these samples by generalizing over templates. Because of the large fraction of rare template samples, the overall performance is strongly dependent on these.

#### Learning from Rare Templates

Next, we analyze the effect on performance of rare template samples in the training set, as opposed to those in the test set. In a classification setting, it is only useful to include classes if they are recurring, i.e., represented by more than one sample. However, in the *USPTO-sm*/*USPTO-lg* datasets, many templates occur only once (see Figure 2). If the templates are modeled as categories, as done in the DNN approach, a large fraction of samples cannot contribute to performance. However, this does not hold for models that can generalize across templates, as our MHN model is able to do. To show the effect of the rare template samples on learning, we use the following experiment on *USPTO-sm*: We removed all samples with templates that are *exactly once* in the training set and *not* in the test set and retrain the best DNN and MHN models of the template relevance prediction experiment. After removal of these samples, the top-10 accuracy rose from 71.2 ± 0.2 to 72.3 ± 0.2 for the DNN+pretrain model and dropped from 78.8 ± 0.4 to 73.7 ± 0.3 for the MHN model. As expected, the performance does not drop for the DNN model, but even improved marginally, which we attribute to the model knowing which templates do not occur in the test set. In contrast, the performance for the MHN model decreased. This shows that the increased performance of our approach is in part caused by the larger fraction of data that can be leveraged for learning.

### Single-Step Retrosynthesis

Next, we compare our method to previously suggested ones in the single-step retrosynthesis task using the *USPTO-50k* and *USPTO-full* datasets. We followed the preprocessing procedure of ref (13) and used rdchiral^63^ to extract reaction templates. Following ref (13), we shuffled the data and assigned 80%/10%/10% of the samples in each reaction class into train/validation/test set, respectively. This is similar to USPTO-sm above but varies in details discussed in section S3. Also, in contrast to template relevance, we optimize for maximum top-1 accuracy, which results in different selected hyperparameters. In addition, we report results of a single run on the *USPTO-full* dataset preprocessed by Tetko et al.^20^ (see Table S4 in the Supporting Information). We first compare the predictive performance of our method to previous ones and then investigate its inference speed.

#### Predictive Performance

Table 2 shows the reactant top-*k* accuracies on *USPTO-50k* for different methods. These methods include, among others, transformer-based,^20,68^ graph-to-graph^67^ or template-based ones.^29^ Some methods^18,25,37,73−77^ that also report results on *USPTO-50k* have been omitted here, either because of test set leakage or different evaluation conditions, as detailed in Section S3. We reimplemented and improved the NeuralSym method as described in Section S3 and added the popularity baseline described in the section entitled “Template Prediction”. Hyperparameter selection on the validation set returned an MHN model with two stacked Hopfield layers, which we refer to as *MHNreact* (see Section S3). We ranked reactant sets by the score of the template used to produce them. If a template execution yielded multiple results, all were included in the prediction in random order. Our method achieved state-of-the-art performance for *k* ≥ 3 and approaches it for *k* = 1, 3. Together with Dual-TB,^66^ this puts template-based methods ahead of other approaches at all considered values of *k*.

**Table 2:**
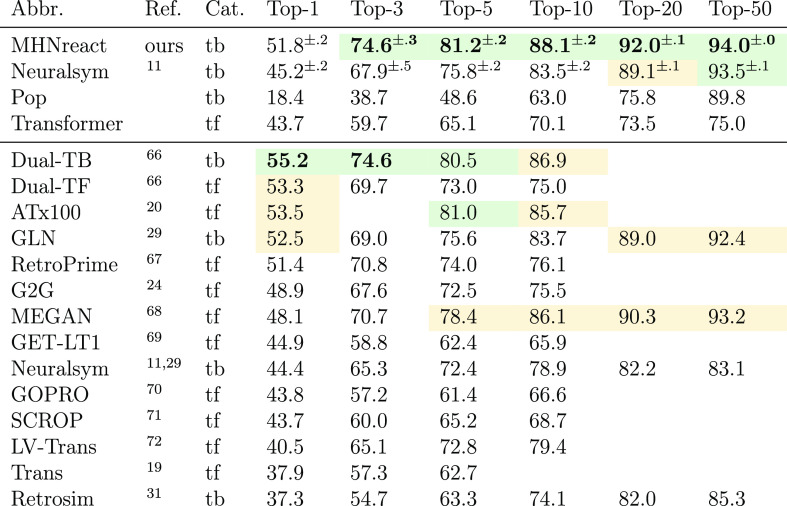
Reactant Top-*k* Accuracy (%) on *USPTO-50k* Retrosynthesis^a^

a Data taken from refs (11, 19, 20, 24, 29, 31, and 66−72). Bold values indicate values within 0.1 of the maximum value, green denotes a value within 1 percentage point of the maximum value, and yellow denotes a value within 3 percentage points to the maximum value. Error bars represent standard deviations across five reruns. Category (“Cat.”) indicates whether a method is template-based (tb) or template-free (tf). Methods in the upper part have been (re-)implemented in this work.

Without the canonicalization procedure of the product-SMILES from the mapped reaction smiles, we obtained a significant increase in performance (Top-1 accuracy of 59.04%). This might suggest leakage, as observed ín ref (73), or signal getting lost from canonicalization procedure. This is apparent when using mini-hash fingerprint (MHFP),^78^ a SMILES-based fingerprint. For all our experiments, we canonicalize the product-SMILES.

### Inference Speed

**Figure 4:**
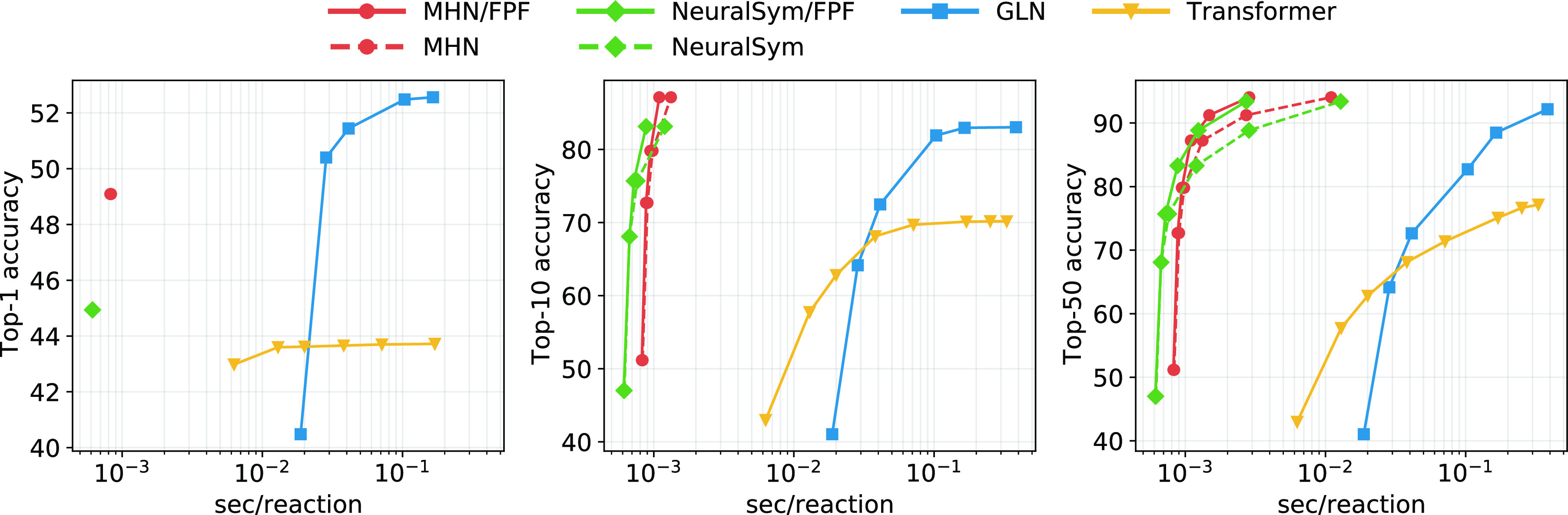
Reactant top-*k* accuracy versus inference speed for different values of *k*. Upper left is better. For Transformer/GLN, the points represent different beam sizes. For MHN/NeuralSym, the points reflect different numbers of generated reactant sets, namely, {1, 3, 5, 10, 20, 50}. In case of a Transformer, the points depict different beam sizes: {1, 3, 5, 10, 20, 50, 75, 100}, from left to right.

Aside from predictive performance, inference speed is also vital for retrosynthesis methods. Therefore, CASP methods are often evaluated by their ability to find a route in a given time.^9,28,79^ Template-based methods are sometimes reported to be slow;^24,68^ however, we found that inference speed was not reported in mentioned studies and generally are seldom reported, despite their importance. Accuracy can be traded for inference speed for many models. For some, this tradeoff is achieved by varying the beam size.^20,29^ In template-based approaches, the number of executed templates can be varied and traded off against speed. We compared inference speed of our MHN method with the following baselines. We obtained results for a graph logic network (GLN) from their paper.^24^ We trained a Transformer baseline using the code of ref (14), as a representative of transformer-based methods.^19,20,72^ In addition, we also include the NeuralSym^11^ model that we implemented in the comparison. The results are displayed in Figure 4. At comparable or better performance, our method achieves inference speed of up to two magnitudes faster, compared to the Transformer and GLN. While NeuralSym is faster than our model for some fixed values of accuracy, MHN yields better maximum accuracy with comparable speed.

### Computation Time and Resources

All experiments were run on different servers with diverse Nvidia GPUs (Titan V 12GB, P40 24 GB, V100 16GB, A100 20GB MIG), using PyTorch 1.6.0.^80^ We estimate the total run time, for all experiments we performed for this study, including baselines, to be ∼1000 GPU hours. A single MHN model can be trained on *USPTO-50k* within ∼5 min on a V100.

## Discussion and Conclusion

In this work, we have reformulated the problem of template-based reaction and retrosynthesis prediction as a retrieval problem. We introduced a deep learning architecture for reaction template prediction, based on using modern Hopfield networks. The proposed architecture consists of a molecule encoder, a reaction template encoder network, and Hopfield layers. The best network architectures that were found during hyperparameter selection are typically relatively lightweight, with one or two stacked Hopfield layers, compared to the sizable Transformer architectures.

The retrieval approach enables generalization across templates, which makes zero-shot learning possible and improves few-shot learning. On the single-step retrosynthesis benchmark *USPTO-50k*, our MHN model reaction reaches the state-of-the-art at top-*k* accuracy for *k* ≥ 3. Furthermore, we falsify the common claim of template-based methods being slow.

We note that the current *USPTO-50k* benchmark and its great emphasis on top-1 accuracy for single-step retrosynthesis might only reflect part of what is needed for single-step retrosynthesis. In cases where a molecule can be made by multiple routes, top-1 accuracy might be too ambiguous; however, the evaluation unrealistically expects to use the one that is present in the dataset. We further argue that there is a tradeoff between having diverse results and having accurate results.^81,82^ Unfortunately, it is currently hard to measure diversity, because of not having multiple correct ground-truth answers per product molecule.

Our experiments are currently limited by several factors. We did not investigate the importance of radius around the reaction center used for template extraction. We currently do not rerank reactants based on a secondary model, such as an in-scope filter^9^ or dual models,^66^ which could increase performance. There would also be several other hyperparameters to be explored, such as the template encoding, whose exploration could lead to an improvement of our method. The results for inference speed are dependent highly on implementation and may potentially be improved by relatively simple means, which was not the primary focus and is left for future work.

Nevertheless, we envision that our approach will be used to improve CASP systems or synthesis-aware generative models.^83−87^

## Supporting Information Available

Additional background information and related work (Section S2); details on experiments (Section S3); and additional results for USPTO-full, as well as, e.g., results for a chronological split for USPTO-50k (Section S4); visualizations on a down projection of the association space of reaction templates (Section S5); showcasing of several exemplary test-set samples of USPTO-50k and a reactant ranking comparison, to illustrate differences between methods (Section S6); an alternative view on the loss and an extended formulation of the algorithm as pseudocode (Section S7); details on experiments (e.g., dataset preprocessing, training, and hyperparameter selection, feature extraction from molecules, as well as details on template-fingerprint featurization can be found in the Supporting Information document (PDF)

## Notes

**Data and Software Availability**: Python code and instructions to reproduce the predictive results of template prediction as well as single-step retrosynthesis are available at https://github.com/ml-jku/mhn-react under the BSD 2-Clause license. Instructions to prepare the programming-environment, as well as to download the data and run the training, inference, and evaluation procedure can be found there. The code was run on different servers with diverse Nvidia GPUs using PyTorch 1.6.0.^80^
