# Supplementary material for: Improving Few- and Zero-Shot Reaction Template Prediction Using Modern Hopfield Networks
Source: J Chem Inf Model. 2022 Jan 15;62(9):2111–20. doi: 10.1021/acs.jcim.1c01065 (PMC9092346; doi:10.1021/acs.jcim.1c01065)
Supplement: Supplementary file 1 — ci1c01065_si_001.pdf [file ci1c01065_si_001.pdf]

Supporting Information for Improving few- and zero-shot reaction template prediction using modern Hopfield networks

Philipp Seidl,[†] Philipp Renz,[†] Natalia Dyubankova,[‡] Paulo Neves,[‡] Jonas Verhoeven,[‡] Jörg K. Wegner,[¶] Marwin Segler,[§] Sepp Hochreiter,[†,‖] and Günter Klambauer[*,†]

†*Johannes Kepler University Linz, ELLIS Unit Linz, LIT AI Lab, Institute for Machine Learning, Altenbergerstraße 69, Linz, AT 4040*

‡*Janssen Pharmaceutica NV, High Dimensional Biology and Discovery Data Sciences, Janssen Research & Development, Turnhoutseweg 30, Beerse, BE 2340*

¶*Janssen Research Development, LLC, In-Silico Discovery and External Innovation (ISDEI), 1 Cambridge Center, 255 Main St, Cambridge, MA 02142, USA*

§*Microsoft Research, 21 Station Road, Cambridge, UK CB1 2FB*

‖*Institute of Advanced Research in Artificial Intelligence, Landstraßer Hauptstraße 5, Wien, AT 1030*

E-mail: klambauer@ml.jku.at

This document provides more related work (see Section S2), then details on the experiments (see Section S3), additional results (see Section S4), visualization (see Section S5), comparison of different methods for selected examples for single-step retrosynthesis (see Section S6)), as well as an alternative view on the loss and an extended formulation of the algorithm as pseudo-code (see Section S7).

Contents

S1 Notation **3**

S2 Further related work **3**

S3 Details on Experiments **4**

S3.1 Template relevance prediction 4

S3.1.1 Datasets and preprocessing 4

S3.1.2 Data splits 5

S3.1.3 Feature extraction 6

S3.1.4 Training 8

S3.1.5 Hyperparameter selection and model architecture 8

S3.2 Single-step retrosynthesis 10

S3.2.1 Datasets and preprocessing 10

S3.2.2 Feature extraction 10

S3.2.3 Hyperparameters and model architecture 11

S3.2.4 Methods omitted from comparison 13

S3.2.5 Inference speed 14

S4 Additional Results **15**

S4.1 Template relevance prediction 15

S4.2 Single-step retrosynthesis 17

S4.2.1 USPTO-full 17

S4.2.2 USPTO-50k further splits 17

S4.2.3 Reactant ranking comparison 17

S5 Hopfield Association Space **27**

S6 Illustrative Example for Single-Step Retrosynthesis **27**

S7 Objective and loss functions **28**

S8 List of Acronyms **32**

References **32**

S1 Notation

Table S1: Symbols and notations used in this paper.

Definition	Symbol/Notation	Dimension
set of reaction templates	$\boldsymbol{T}$	set of size K
encoded set of reaction template	$\boldsymbol{T}_h$	$d_t \times K$
reactant molecules	$\boldsymbol{r}$	
product molecule	$\boldsymbol{m}$	
encoded molecule	$\boldsymbol{m}_h$	d_m
reaction template	$\boldsymbol{t}$ or $\boldsymbol{t}_k$	
training set pair	$(\boldsymbol{m}, \boldsymbol{t})$	
state pattern	$\boldsymbol{\xi}$	d
stored pattern	$\boldsymbol{x}_k$	d
stored pattern matrix	$\boldsymbol{X}$	$d \times K$
associations	$\boldsymbol{p}$	K
update function of modern Hopfield network (MHN)	f	
molecule encoder	$\boldsymbol{h}^m$	
reaction template encoder	$\boldsymbol{h}^t$	
model function	g	
network parameters of $\boldsymbol{h}^m$	$\boldsymbol{w}$	
network parameters of $\boldsymbol{h}^t$	$\boldsymbol{v}$	
parameters of Hopfield layer $\boldsymbol{h}^m$	$\boldsymbol{W}_m, \boldsymbol{W}_t$	$d \times$ undef
association activation function	ϕ	
number of templates	K	
dimension of association space	d	

S2 Further related work

Here we provide a broader view on works that have addressed common issues with template relevance prediction. In prior work[1–6], template relevance prediction is often viewed as a multi-class classification task, where, given a product, an machine learning (ML) model is trained to predict which of the templates extracted from a reaction database are most relevant. Automatic extraction of templates leads to many rare templates, which poses a problem in the classification task as it leads to many classes with few training samples[3,5]. In earlier work[5], rare templates were excluded from training. Baylon et al.[1] proposed a hierarchical grouping

of reaction templates and trained a separate NN for each group of templates. Fortunato et al.[3] pre-trained their template scoring model to predict which templates are applicable and observed that it improves template-relevance predictions, especially for rare templates.[2] used a graph-NN to predict the relevant templates. Bjerrum et al.[6] trained two separate NNs. The first NN is trained on the applicability matrix and serves for pre-filtering reaction templates. The second is trained on the reaction dataset and ranks the reaction templates according to their relevance. Dai et al.[7] make use of the template structures. However, they factorize the predicted probabilities into multiple functions, which might not be suited to the problem. The in-scope filter of the computer-assisted synthesis planning (CASP) system by Segler et al.[5] also make use of template structure. Sun et al.[8] apply all templates and uses a model to rank the resulting reactants, which achieves the best top-1 accuracy but is computationally costly (Dual-TB in Table 2).

S3 Details on Experiments

S3.1 Template relevance prediction

S3.1.1 Datasets and preprocessing

For preprocessing USPTO-sm and USPTO-lg, we followed the implementation of Fortunato et al.[3]. The templates were extracted from the mapped reactions using RDChiral[9] and subsequently filtered according to symmetry, validity, and by checking if the application of the template yielded the result as in the reaction the template originated from. Despite adhering to the original implementation by the authors, our preprocessing resulted in different dataset sizes. The roughly 2 million starting reactions decreased to 443,763 samples and 236,053 reaction templates for USPTO-lg (compared to 669,683 samples and 186,822 reaction templates in Fortunato et al.[3]), and to 40,257 samples and 9,162 reaction templates for USPTO-sm (compared to 32,099 samples and 7,765 reaction templates in Fortunato et al.[3]).

It can be seen that 17% of samples occur in a class that has only one sample in USPTO-sm and 43% in USPTO-lg. 66% of reaction templates in USPTO-sm and 80% in USPTO-lg occur only in a single reaction.

To allow for pre-training of the methods, we calculated the applicability matrix, i.e., which templates in a dataset are applicable to which molecules. Fortunato et al. [3] reported that this would take ∼36 CPU-hours for USPTO-sm and ∼330 CPU-days for USPTO-lg on a single core of a Xeon(R) Gold 6154 CPU@3 GHz. We found that using a substructure screen could speed up this procedure. The following values were obtained using an AMD EPYC 7542. For USPTO-sm it only takes us 3.3 CPU-minutes to achieve the same result. Using 16 CPU-cores this reduces to $\sim 14s$. Using 32 CPU-cores applicability calculation time for USPTO-lg reduces to $\sim 50m$ which corresponds to 27 CPU-hours compared to ∼8000 CPU-hours for the original implementation. These comparisons should be taken with a grain of salt because of the slightly different dataset sizes and hardware used. For USPTO-lg $443\,763 \cdot 236\,053 \sim 10^{11}$ pairs have to be checked, and our code relies on a python loop. Using a compiled language could probably further speed up the procedure.

S3.1.2 Data splits

We split the data into a training, validation, and test set following Fortunato et al. [3]. Here, a stratified split was used to ensure that templates are more equally represented across the splits. Concretely, in Fortunato et al. [3], the split proportions were 80/10/10% except for templates with fewer than 10 samples, where one random sample was put into the test set, one into the validation set, and the rest into the training set. If only two samples were present, one was put into the test and one into the training set. Finally, if only a single sample was available for a template, it was randomly placed into the train/validation/test set with an 80/10/10% chance.

S3.1.3 Feature extraction

Molecule fingerprints. The source molecules are represented as simplified molecular-input line-entry system (SMILES). We extract a fingerprint representation of the molecules. A molecular fingerprint represents the absence and presence of substructures within a molecule. The way those subgraphs or substructures are selected is very much different and depends on the chosen method as well as their parameters. For example, when using morgan fingerprints[10], the algorithm iterates through each atom in the graph, and notes all substructure up to a certain radius. All substructures are hashed to each map to an integer. All integers are folded (modulo operation) to a fixed length. The longer the less collisions will appear. The final vector is a rather efficient indication of how many substructures two molecules share, and therefore one way to measure their similarity.

We tried out different fingerprint types, e.g. folded Morgan fingerprints with chirality[10] and the hyperparameter selection procedure (see Table S2) selected Morgan fingerprint with radius of 2 folded to 4096 features.

Template fingerprints. For the template representation, a similar procedure has been applied. A template consists of multiple enumerated SMILES arbitrary target specification (SMARTS)-strings. The fingerprint type for the templates was set to `rdk`-fingerprint or `pattern`-fingerprint for template relevance prediction and calculated for each molecule that the pattern represents. We experimented with multiple ways of combining not just the product side, but also the reactant side to this representation. We found the following to perform best among the considered variants. The fingerprints were calculated for each molecular pattern, and a disjunction over reactants as well as products was calculated. The product minus half of the reactant side results in the template fingerprint as input for the template encoder. We also tried the `structuralFingerprintForReaction` function provided by RDKit[11], which concatenates the disjunction of each side of the reaction, but found the weighted combination to perform better.

Consider a template consisting of reactant substructures s_r and product substructure

s_p. Using a fingerprint-function f_p produces a fingerprint-vector $x_{r_1} \ldots x_{r_n}$ and x_p. The final template fingerprint x_t is computed using the following form:

$$x_t = f_p(s_p) - \cdot 0.5 \text{pool}(f_p(s_{r_1}), ..., f_p(s_{r_n}))$$

where pool is a pooling function. A few things need to be considered for the implementation as can be found in the linked github repository. To compute the fingerprints for each of the substructures the function `getFingerprint` from `molutils` is used and `issmarts` is set to True. By default the fingerprint-size, -type, or -radius (if applicable) is defined by the hyperparameters. Sanitization is an important step and done by default. Here the `SanitizeMol` function from RDKit[11] is employed setting `catchErrors` parameter to True. The next step is using the `FastFindRings` function to providing ring information if applicable. Further `UpdatePropertyCache(strict=False)` to correcting valence information is used. Max-pooling is used as pooling operation. Another thing to note is that, e.g. number of explicit hydrogens, direct bonds or charge, may be present in a SMARTS-template, but will not be represented by the fingerprint in RDKit. Nevertheless, the resulting representation allows for a learnable measure of similarity between templates, but for some templates the representation turned out to be equal, and therefore indistinguishable. A simple, yet effective approach is to add an additional template embedding, which adds more flexibility and has less inductive bias.

The randomly initialized embedding was added to the representation of frequent templates in order to help to differentiate frequent templates with a high fingerprint similarity. Templates are classified as frequent if they appear at least a certain number of times in the training-set, which is determined by the hyperparameter `random template threshold` (see Table S2).

S3.1.4 Training

All models were trained for a maximum of 100 epochs on a Titan V with 12 GB RAM or a P40 with 24 GB RAM using PyTorch 1.6.0[12]. In the case of deep neural network (DNN), only the molecule encoder was trained, and a linear layer, projecting from the last hidden layer to the number of templates was added. For pre-training on the applicability task we changed the loss function to the mean of binary cross-entropy for each output (template). We also experimented with Information Noise-Contrastive Estimation (InfoNCE)-loss[13] on representations in Hopfield space (see S7). Because of fast convergence and slightly better performance, and because for the United States patent and trademark office (USPTO) data sets only a single template is correct for each molecule, we use our proposed loss, which in this case is equivalent to CE-loss.

S3.1.5 Hyperparameter selection and model architecture

Hyperparameters were explored via automatic Bayesian optimization for USPTO-sm, as well as manual hyperparameter-tuning. In the former, early stopping was employed. The range of values was selected based on prior knowledge. Additional manual hyperparameter-tuning resulted in better predictive performance on the validation set. Some of the important hyperparameters are the beta scaling factor of the Hopfield layer β, the dimension of the association space d, as well as the association-activation function, or if the association space should be normalized via layer-norm[14]. An overview of considered and selected hyperparameters is given in Tab. S2. All models were trained if applicable for a maximum of 100 epochs using AdamW[15] (`betas`=(0.9, 0.999), `eps`=1e−8, `weight_decay`=1e−2, `amsgrad`=False). Hyperparameters were selected based on the minimal CE-loss on the validation set.

Table S2: Hyperparameter search-space for template relevance prediction. The rows are subdivided into five modules: overall parameters, the molecule encoder, the template encoder, the Hopfield layer, and setting specific hyperparameters that were only used if explicitly stated. The column values show the range of the explored parameters. If multiple Hopfield layers were used, the same hyperparameters were used for all layers. A `random template threshold` of `-1` corresponds to not adding noise. The fingerprint size for the molecule encoder was always the same as the template encoder. The pre-training learning rate was also defined by the learning rate, the optimizer remained the same, but the loss-function changed to binary-cross-entropy loss.

**Hyperparam**	**Values**	**MHN selected (Sm/Lg)**	**DNN selected (Sm/Lg)**
learning rates	{1e-4, 2e-4, 5e-4, 1e-3}	5e-4 / 1e-4	5e-4 / 2e-4
batch-size	{32, 128, 256, 1024}	1024	256 / 1024
dropout	[0.0, 0.6]	0.2	0.15
molecule encoder			
fingerprint type	{morgan, rdk}	morgan	morgan
fingerprint size	{1024, 2048, 4096}	4096	4096
number of layers	{0, 1, 2}	0	1
layer-dimension	{1024, 2048, 4096}	-	2048
activation-function	{None, SELU, ReLU}	None	ReLU
template encoder			
number of layers	{0, 1, 2}	0	
template fingerprint type	{pattern, rdk}	rdk	
random template thresh.	-1, 2, 5, 10, 50	2	
Hopfield layer			
beta	[0.01, 0.3]	0.03	
association af	{None, SELU, GeLU, Tanh}	None / Tanh	
normalize pattern	{False, True}	False	
normalize projection	{False, True}	True	
learnable stored-pattern	{False, True}	False	
hopf-num-layers	{1, 2, 3}	1	
hopf-num-Wm	{1, 2, 3}	1	
hopf-num-Wt	{1, 2, 3}	1	
hopf-FF-activation	{None, SELU, ReLU}	None	
association-dimension d	{32, 64, 512, 1024}	1024	
hopf-num-heads	{1, 6, 12}	1	
Setting-specific-hps			
pre-training epochs	{0,5,10,15,20,25}	10	25 / 5

S3.2 Single-step retrosynthesis

S3.2.1 Datasets and preprocessing

For single-step retrosynthesis, we used the preprocessed version and splitting-procedure from Coley et al.[16]. The dataset originated from USPTO-50k by Schneider et al.[17]. It is different in details from USPTO-sm and does not contain a filtering step, whereby samples are excluded if extracted and applied templates don't yield the reactants. A further difference is the split. For USPTO-50k, we shuffle the samples and further split it according to the procedure by Coley et al.[16], randomly splitting within the reaction types, to obtain 40008 train- 5001 validation- and 5007 test-samples (80/10/10). We computed the reaction templates only from the train- and validation-set.

We additionally report results for USPTO-full which was preprocessed and split randomly by Tetko et al.[18]. The dataset contains almost 1M samples (train: 761.335, valid: 95.181, test: 96.027), and extracting templates from the train and validation set lead to 257.340 templates. Note that there are less test samples as reported in Dai et al.[7] due to the different pre-processing and removal of duplicate reactions.

S3.2.2 Feature extraction

For this experiment, we additionally explored Mixed-Fingerprint (MxFP), which is a mixture of multiple folded, counted (where applicable) fingerprints: molecular access system (MACCS), Morgan, ErG, AtomPair, TopolocialTorsion, mini-hash fingerprint (MHFP)[19] and RDK. We additionally scale the counts by $\log(1 + x)$[5].

Template-representation. We compute fingerprints for each subgraph-pattern in the reaction template. Again we use a mixture of multiple unfolded RDKit fingerprints. For pooling the reactant fingerprints, we additionally experimented with different pooling operations. The main idea is to avoid that different sets are identical after pooling and thus to increase the expressivity of the pooling operation. Lgamma pooling is a novel pooling operation that

uses the log of the gamma-function.

$$\mathrm{lgp}(\boldsymbol{x}) = \log\left(\Gamma\left(\sum_{i=0}^{n} x_i + 2\right)\right) - \sum_{i=0}^{n} \log(\Gamma(x_i + 1)), \tag{1}$$

where the $\boldsymbol{x}$ contains a single feature of the elements of the set that is pooled. The use of this pooling function provided a small performance increase over max-pooling.

S3.2.3 Hyperparameters and model architecture

Hyperparameters were tuned manually and selected based on top-1 accuracy on the validation set. The explored parameters, as well as the selected hyperparameters, can be found in Table S3. Models were also trained if applicable for a maximum of 100 epochs using AdamW[15] (betas=(0.9,0.999), eps=1e-8, weight_decay=1e-2, amsgrad=False). As input, MxFP was selected with a fingerprint size of 30k. It consists of two layers, where the input for the second layer is $\boldsymbol{\xi}^{\text{new}} + \boldsymbol{\xi}$, a skip connection from the first layers input plus the output of the first layer. The first layers' memory is comprised of MxFP-template fingerprints with lgamma-pooled reactants (see Section S3.2.2). The second layer uses a different template representation: RDK-template fingerprint with additional random noise for all templates which appear more than once in the training set. The final prediction is computed by a weighted average of the individual layers' $\boldsymbol{p}$.

The NeuralSym baseline was trained as follows: As a model architecture, we used a feed-forward neural network with a single hidden layer of size 4096 and self-normalizing linear unit (SELU) activation function.[20] The inputs to this network were extended connectivity fingerprint (ECFP)-fingerprints[21] with radius 2 and size 4096. The model was trained using AdamW[15] with learning rate 1e-3 and weight-decay of 1e-3. The model was trained for 7 epochs with a batch size of 512.

Table S3: Hyperparameter search-space for single-step retrosynthesis. The layout and specifics are equivalent to Table S2 but differ in the explored values and architectural choices. The hyperparameters for the Hopfield layer remain the same among layers, with individually initialized weight parameters. The input for layer 1 is given by "template encoder 1" and vice versa for layer 2. The column MHN (50k/full) corresponds to the results of MHNreact and DNN (50k) to the first mention of Neuralsym in Table 2.

**Hyperparam**	**Values**	**MHN (50k/full)**	**DNN (50k)**
learning rates	$\{1e\text{-}4, 2e\text{-}4, 5e\text{-}4, 1e\text{-}3\}$	5e-4 / 1e-4	1e-4
batch-size	$\{32, 128, 256, 1024\}$	1024	256
dropout	$[0.0, 0.6]$	0.4/0.2	0.15
molecule encoder			
fingerprint type	{morgan, rdk, MxFP}	MxFP	morgan
fingerprint size	$\{4096, ..., 40e3\}$	3e4/2.4e4	4096
fingerprint radius	$\{2, ..., 6\}$	-	2
number of layers	{0, 1, 2}	0	1
layer-dimension	{1024, 2048, 4096}	-	4096
activation-function (af)	{None, SELU, ReLU}	None	SELU
template encoder 1			
number of layers	{0, 1, 2}	0	
template fingerprint type	{rdk, rdkc, MxFP}	MxFP	
random template threshold	-1, 2, 5, 10, 50	-1/2	
reactant pooling	{max, sum, mean, lgamma}	lgamma	
template encoder 2			
number of layers	{0, 1, 2}	0 / -	
template fingerprint type	{rdk, MxFP}	rdk/-	
random template threshold	-1, 2, 5, 10, 50	2/-	
Hopfield layer 1 and 2			
beta	$[0.01, 0.3]$	0.03	
association af	{None, Tanh}	None	
normalize input pattern	{False, True}	True	
normalize association proj.	{False, True}	True	
learnable stored-pattern	{False}	False	
hopf-num-layers	{1, 2}	2/1	
hopf-num-Wm	{1, 2}	1	
hopf-num-Wt	{1, 2}	1	
hopf-FF-af	{None, SELU, ReLU}	None	
association-dimension d	{64, ..., 2048}	1024/1500	
hopf-num-heads	$\{1, 6, 12\}$	1	

S3.2.4 Methods omitted from comparison

We omitted some studies from the comparison in Table 2, despite them reporting performance on USPTO-50k. We found that the experimental settings or reported metrics in these studies differed from ours. While these reported values are not per se flawed, we think that inclusion in the comparison may be misleading. We list specifics below:

- Yan et al.[22] reported (`https://github.com/uta-smile/RetroXpert`) that their model used information in the atom-mappings about where the reaction center is. This information relies on the knowledge of the reactants. As the reactants are to be predicted in this task this is considered test set leakage.

- The reported values in[23] are also based on unintentional use of information about the reaction center, similar to above[1].

- The method proposed in[24] selects reactants from a candidate set. Since this candidate set is a superset of the reactants in the USPTO-50k, it might contain information about the test data. Indeed we found that we could augment the performance of our method by a process of elimination, i.e., discarding reactant sets from the predictions if they are not in the candidate set.

- The method proposed in[25] also relies on a candidate set that we suspect to contain information about the test set. However, the description of the method is not very detailed.

- Guo et al.[26] use reactants from USPTO-stereo as described in[27] as a candidate set. We found that, given this set, we could augment the performance of our method by removing reactant sets not in this set from our predictions.

- Ishiguro et al.[28] propose a pre-training step on a larger data set which does not conform to the setting in most prior work and is therefor excluded.

[1]Personal communication with the authors

- Ishida et al.[2] use a different subset of USPTO-50k to train their model and report different metrics.

- Ucak et al.[29] also make use of a different subset and also do not report reactant top-k accuracy.

- Liu et al.[30] only provide results for the special case where the type of reaction is provided to the model.

S3.2.5 Inference speed

We investigated the speed/performance trade-off for multiple methods. Firstly, we trained a Transformer baseline using the code and settings provided by[27], except for setting the batch size to 8192, warm-up steps to 6k, and train steps to 50k. We evaluated the predictions of this model when run with beam sizes $\{1, 3, 5, 10, 20, 50, 75, 100\}$. While performance increases with larger beam size, the model also gets slower. This model outperforms the model suggested in[31], but could not reach the performance of[18]. Model training took about six hours on an Nvidia V100.

For MHN and NeuralSym, the inference procedure contains the following steps. First fingerprints for the given products have are generated. Then the model is used to predict template relevance. For each product templates are executed in the order of their score until a fixed number of reactant sets are obtained. To optimize top-k accuracy, it does not help to generate more than k reactant sets. Therefore we set the number of reactant sets to generate to $\{1, 3, 5, 10, 20, 50\}$ optimize speed without loss of top-k accuracy for the respective k and measured inference time. Speeds for the Transformer, NeuralSym, and MHN models have been measured using an Nvidia Tesla T4 and 16 cores of an AMD EPYC 7542. We also tested stopping template execution based on the cumulative probability of already executed ones as done in[5], however found that it did not improve upon stopping after a certain number of reactants have been retrieved.

S4 Additional Results

S4.1 Template relevance prediction

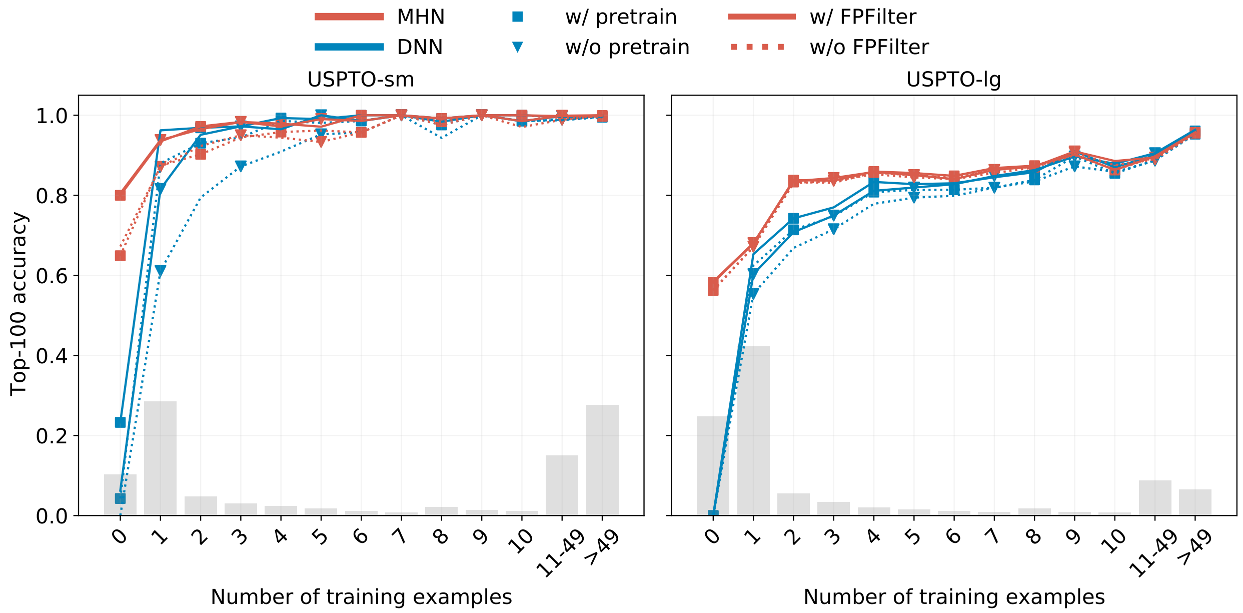

Figure S1: Results of methods with different design elements on the USPTO-sm and USPTO-lg datasets. Each method consists of a combination of the following elements: a) a network MHN or DNN (blue or red line), b) whether pre-training is applied (squares or triangles), and c) whether fingerprint filter (FPF) is applied for postprocessing (solid or dashed line). These eight possible combinations are displayed as lines with their top-100 accuracy on the y-axis and the different template frequency categories on the x-axis.

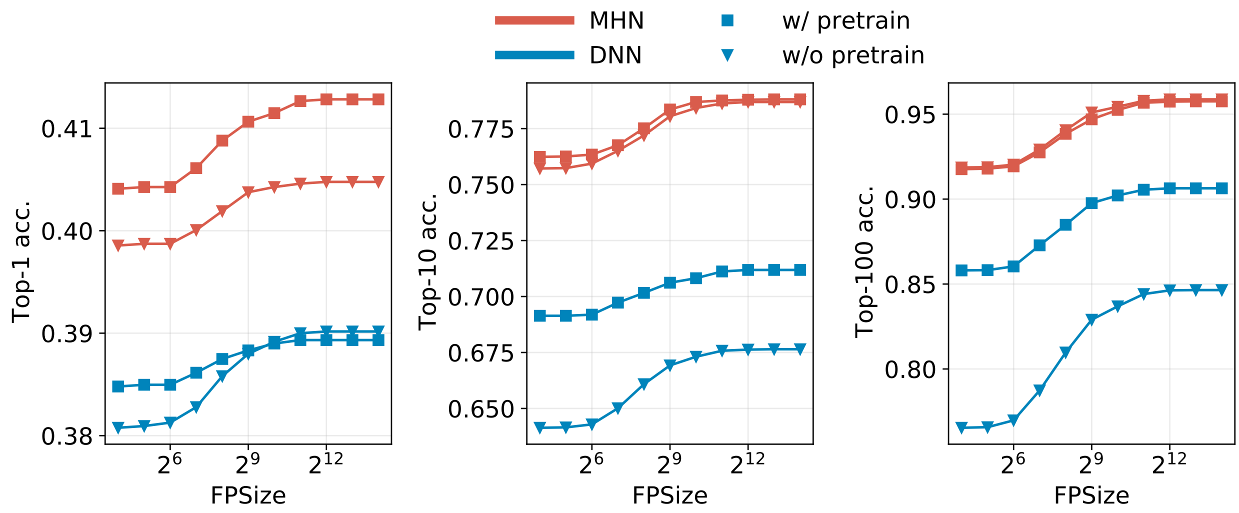

Figure S2: Predictive performance of different methods in dependency of the fingerprint size. Each method consists of a combination of the following elements: a) a network MHN or DNN (blue or red line), and b) whether pre-training is applied (squares or triangles). These four possible combinations are displayed as lines with their top-1, top-10 and top-100 accuracy. Performance saturates at a fingerprint size of about 2^{12}=4096, and we therefore choose this value for the other experiments.

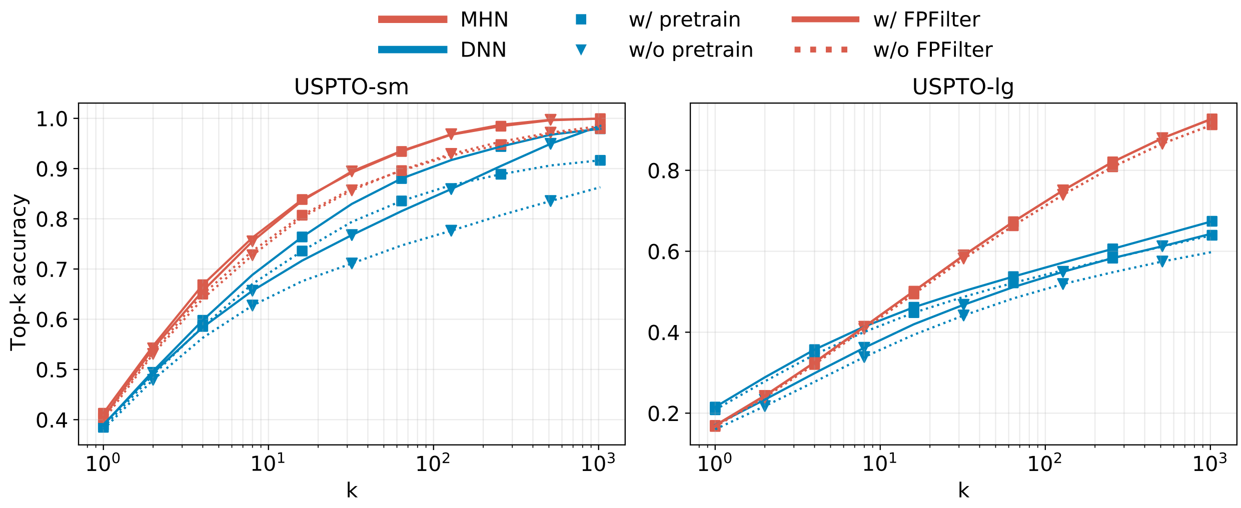

Figure S3: Comparison of methods with respect to their top-k accuracies. Each method consists of a combination of the following elements: a) a network MHN or DNN (blue or red line), b) whether pre-training is applied (squares or triangles), and c) whether FPF is applied for postprocessing (solid or dashed line). These eight possible combinations are displayed as lines with their k parameter on the x-axis and their top-k accuracy on the y-axis. MHNs provide the best top-k accuracy with k larger or equal 10.

S4.2 Single-step retrosynthesis

S4.2.1 USPTO-full

Table S4: Reactant top-k accuracy (%) on USPTO-full retrosynthesis. Bold values indicate values within 0.1, green 1 and yellow within 3 percentage points to the maximum value. Category ("Cat.") indicates whether a method is template-based (tb) or template-free (tf). Methods in the upper part evalute on a version of USPTO-full with pre-processing according to Tetko et al.[18], whereas the lower part has a larger test set (some duplicates), different pre-processing and split as described in Dai et al.[7].

Abbr.	Ref.	Cat.	Top-1	Top-2	Top-3	Top-5	Top-10	Top-20	Top-50
MHNreact	ours	tb	45.5	**57.2**	**62.5**	**67.4**	71.9	**74.8**	**77.1**
ATx100	[18]	tf	**46.2**	**57.2**			**73.3**		
RetroPrime	[32]	tf	44.1		59.1	62.8	68.5		
GLN	[7]	tb	39.3				63.7		
MEGAN	[33]	tf	33.6				63.9		74.1
Neuralsym	[4,7]	tb	35.8				60.8		
Retrosim	[16]	tb	32.8				56.1		

S4.2.2 USPTO-50k further splits

Table S5: Different split-scenarios for reactant top-k accuracy (%) on USPTO-50k retrosynthesis for MHNreact. Chronological split refers to a split where one trains on data <=2012, validations on 2013 and tests on samples >2013 on a proportion of approx. 78:7:15. Dates have been obtained where possible, through matching USPTO-50k reaction-id's with the original dataset[34] (For 19 samples the year could not be determined). Note that the hyperparameters have been determined on the random split, and not on each split individually.

Split	Top-1	Top-3	Top-5	Top-10	Top-20	Top-50
default random split	$51.8^{\pm.2}$	$74.6^{\pm.3}$	$81.2^{\pm.2}$	$88.1^{\pm.2}$	$92.0^{\pm.1}$	$94.0^{\pm.0}$
10-fold cross-validation	$50.7^{\pm.6}$	$73.2^{\pm.6}$	$80.7^{\pm.4}$	$87.2^{\pm.5}$	$91.1^{\pm.5}$	$93.5^{\pm.5}$
chronological split	43.3	65.9	74.9	82.9	88.4	92.2

S4.2.3 Reactant ranking comparison

In order to illustrate and compare the novelty and effectiveness of our method, we showcase results of different methods, for the same synthesis goal. The ranking, and therefore preferred synthesis-starting point is shown for Top-1 in the left column from Figure S5 to Figure

S12. The results of 3 Methods are being compared: MHNreact, DNN corresponding to Segler and Waller[4] trained using the MHNreact framework with the same input and our re-implementation of a Transformer for single-step retrosynthesis[31].

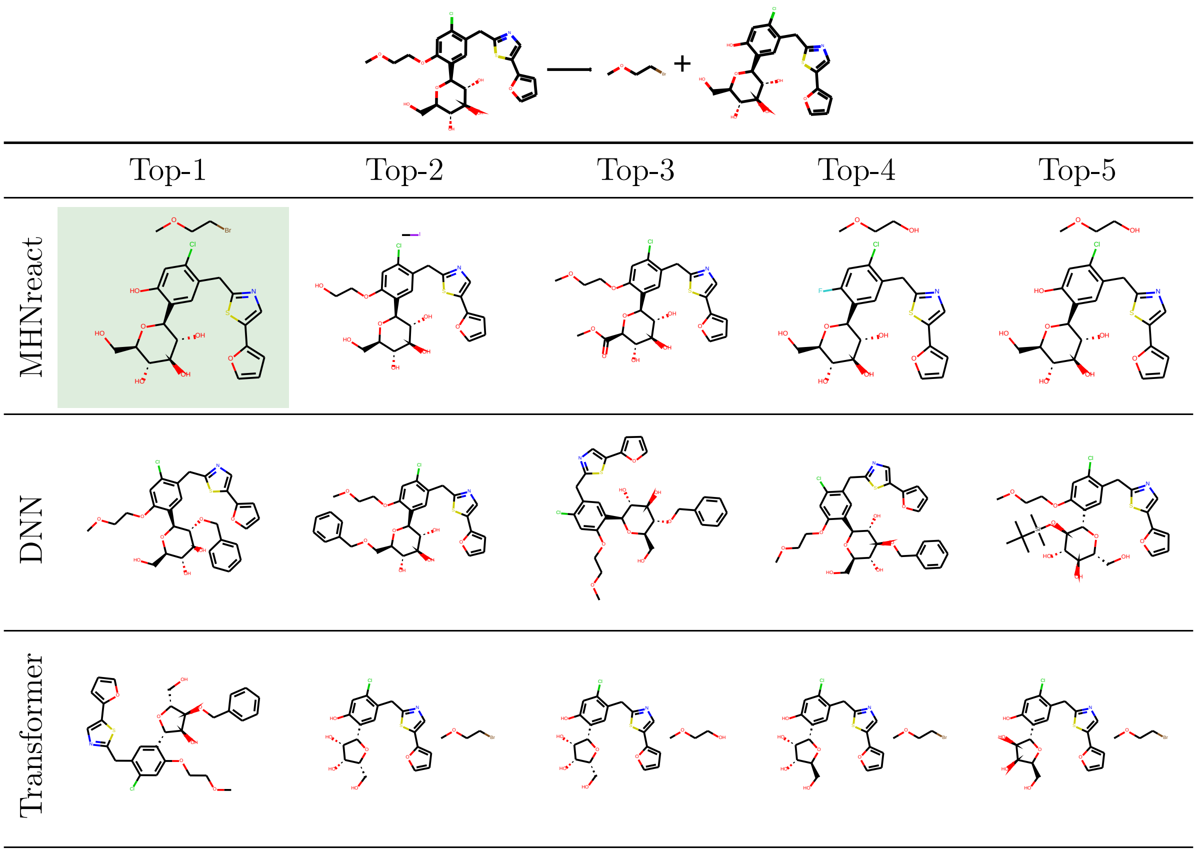

Figure S4: Sample #1117 from the test-set: Top-ranked molecules of different approaches. On the bottom, the ground truth reaction from the dataset is shown, and the matching predicted reactants are highlighted in green. Note that the transformer model makes a copy error, and changes the tetrahydropyran in the start molecule into a tetrahydrofuran.

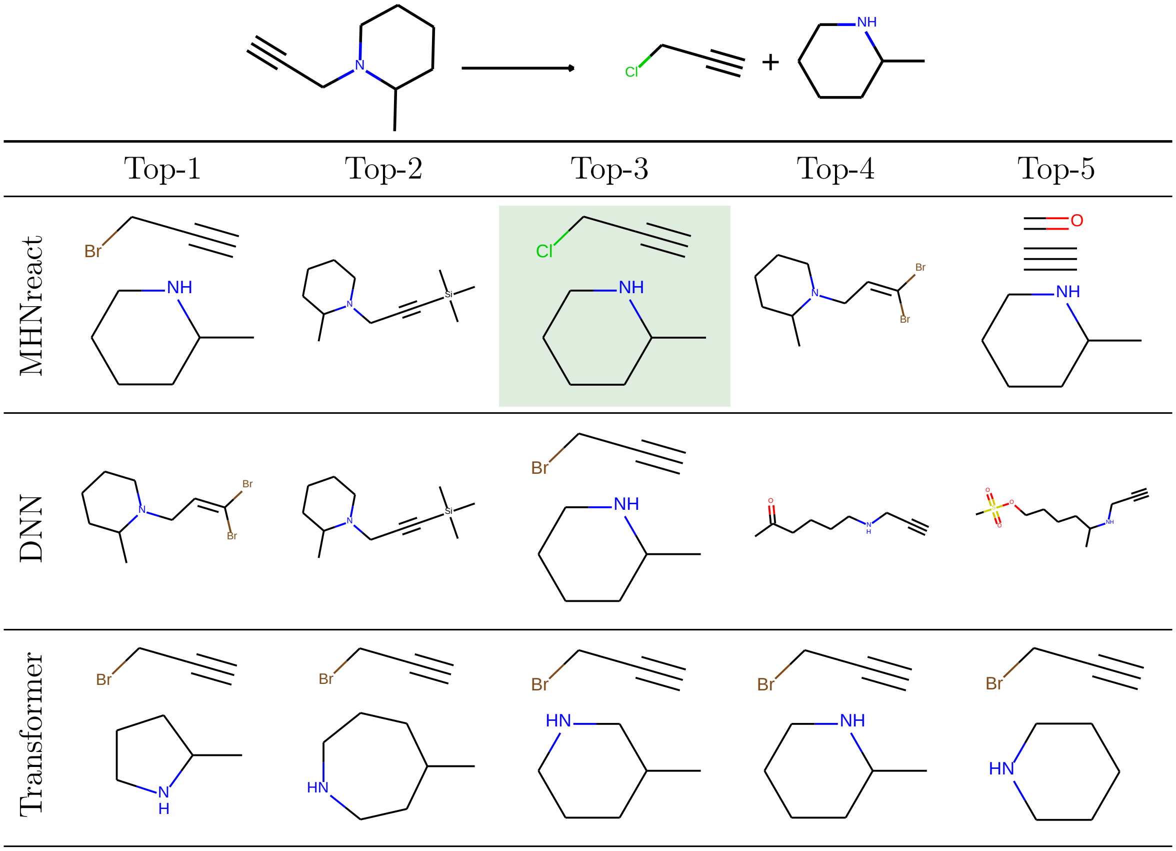

Figure S5: Sample #29 from the test-set. The transformer model exhibits copy mistakes, where the ring size of the piperidine 6-membered ring is changed to a pyrrolidine (5-membered) or azepane (7-membered) ring, or shifts the relative position of the methyl group around.

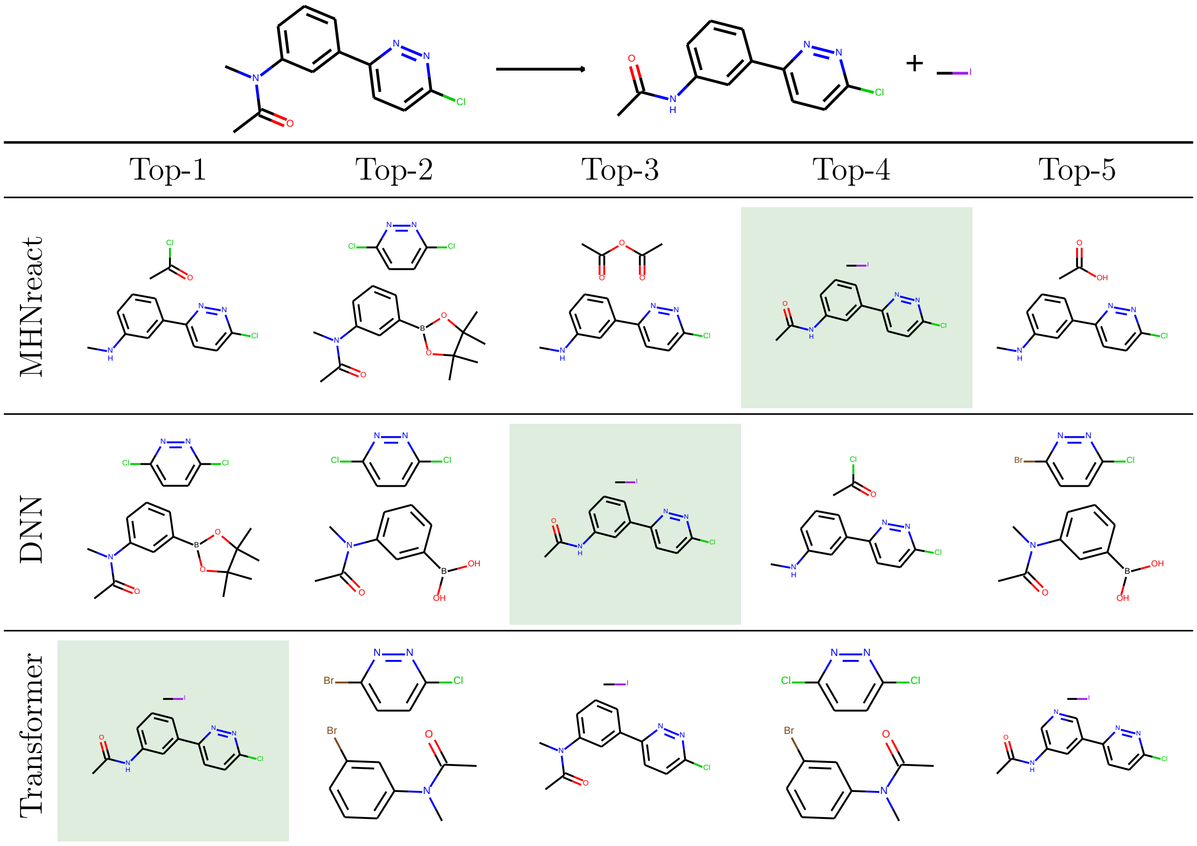

Figure S6: Sample #42 from the test-set.

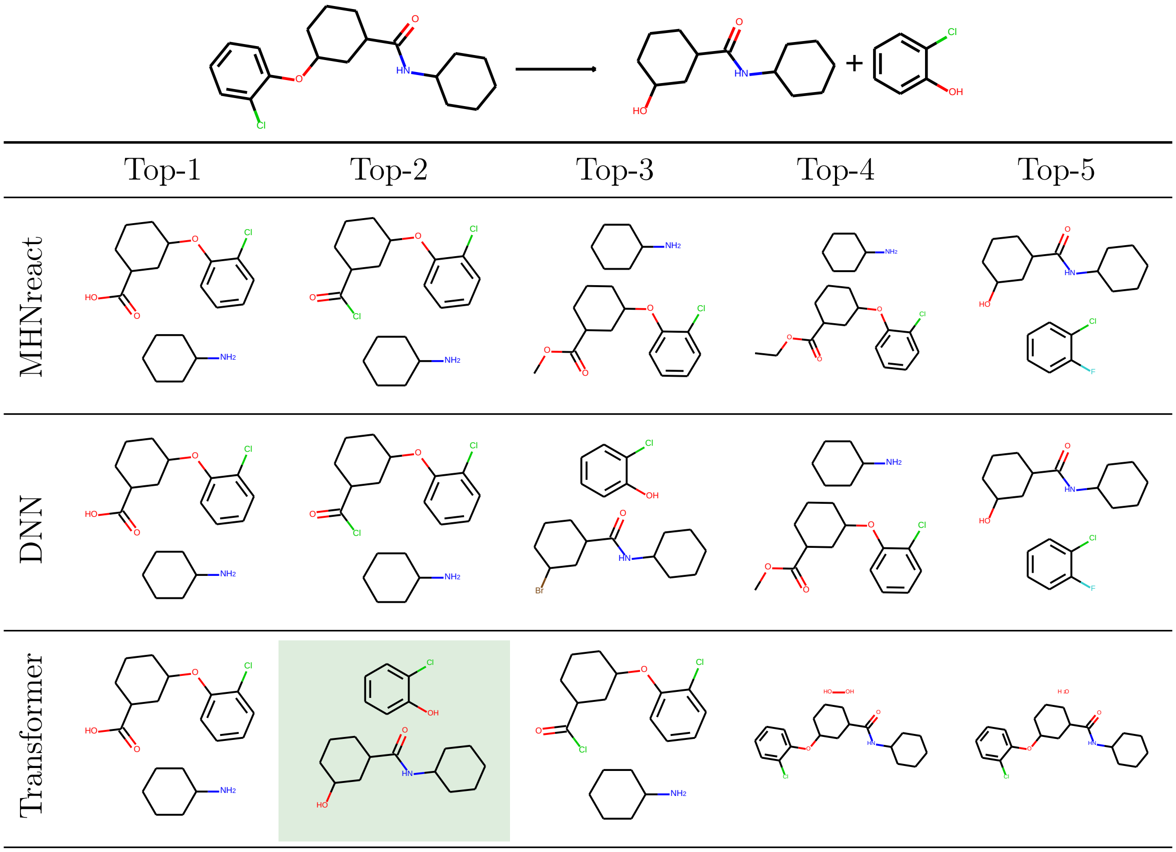

Figure S7: Sample #23 from the test-set.

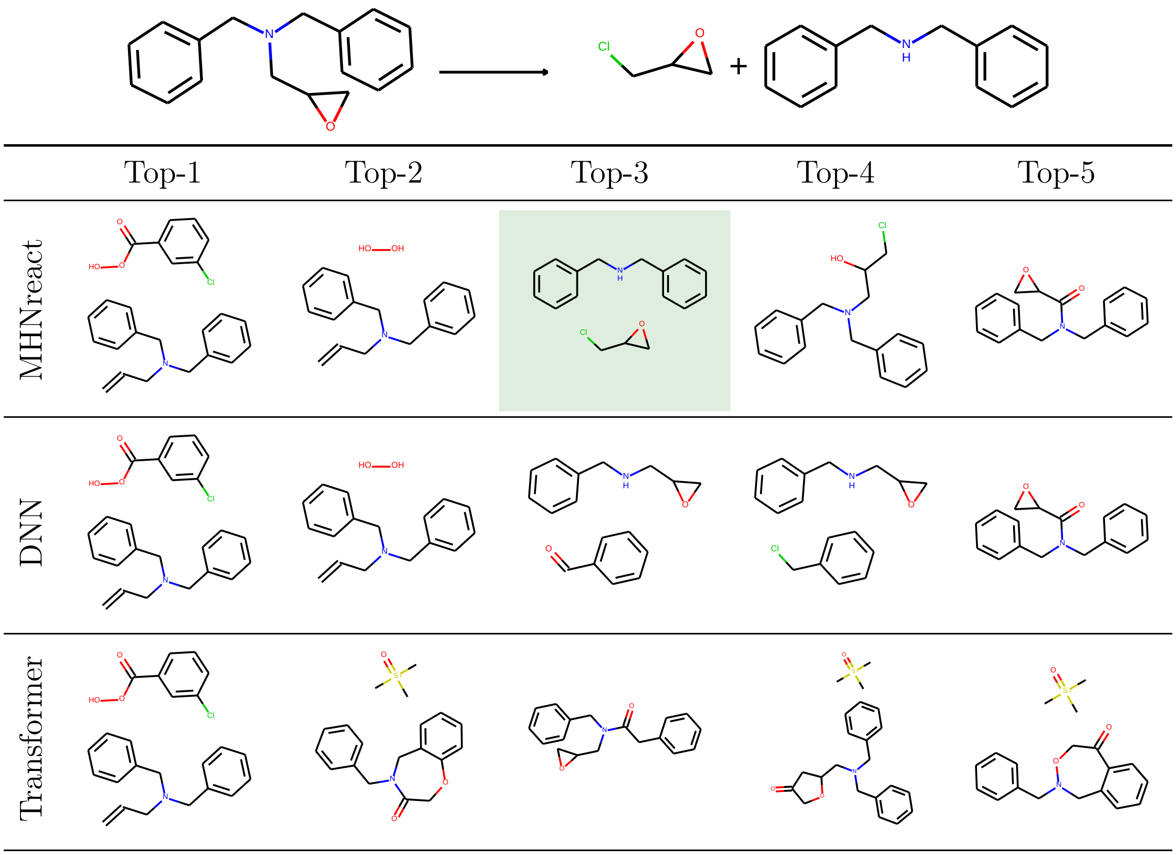

Figure S8: Sample #175 from the test-set.

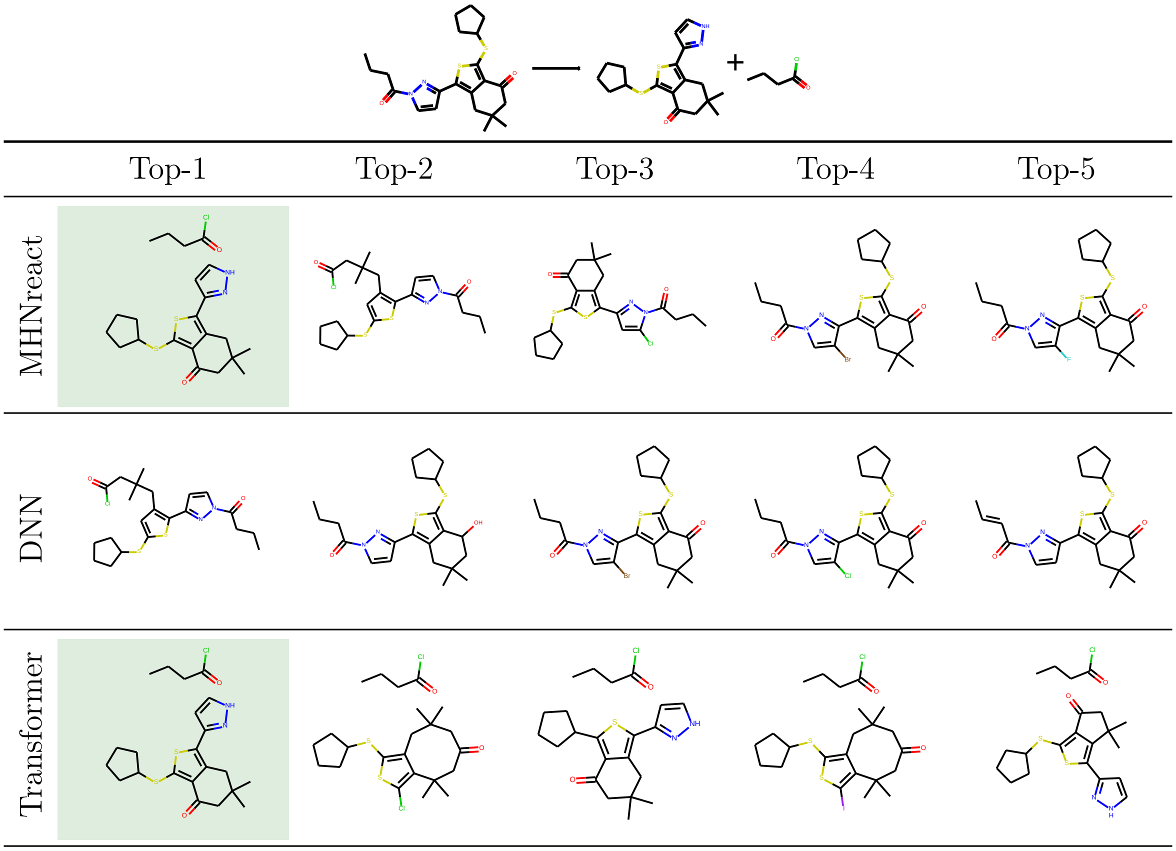

Figure S9: Sample #305 from the test-set. Note the copy errors in the Transformer, leading to ring expansion or contraction on the annelated ring

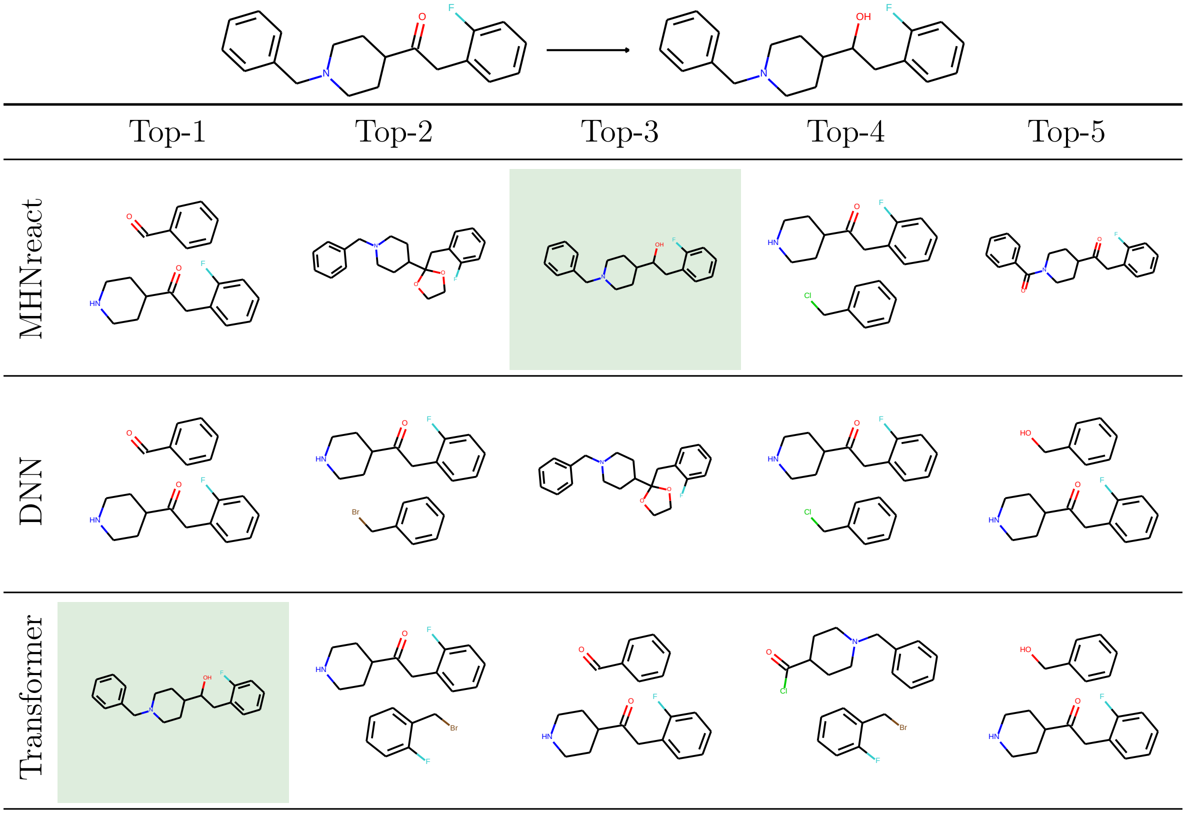

Figure S10: Sample #329 from the test-set.

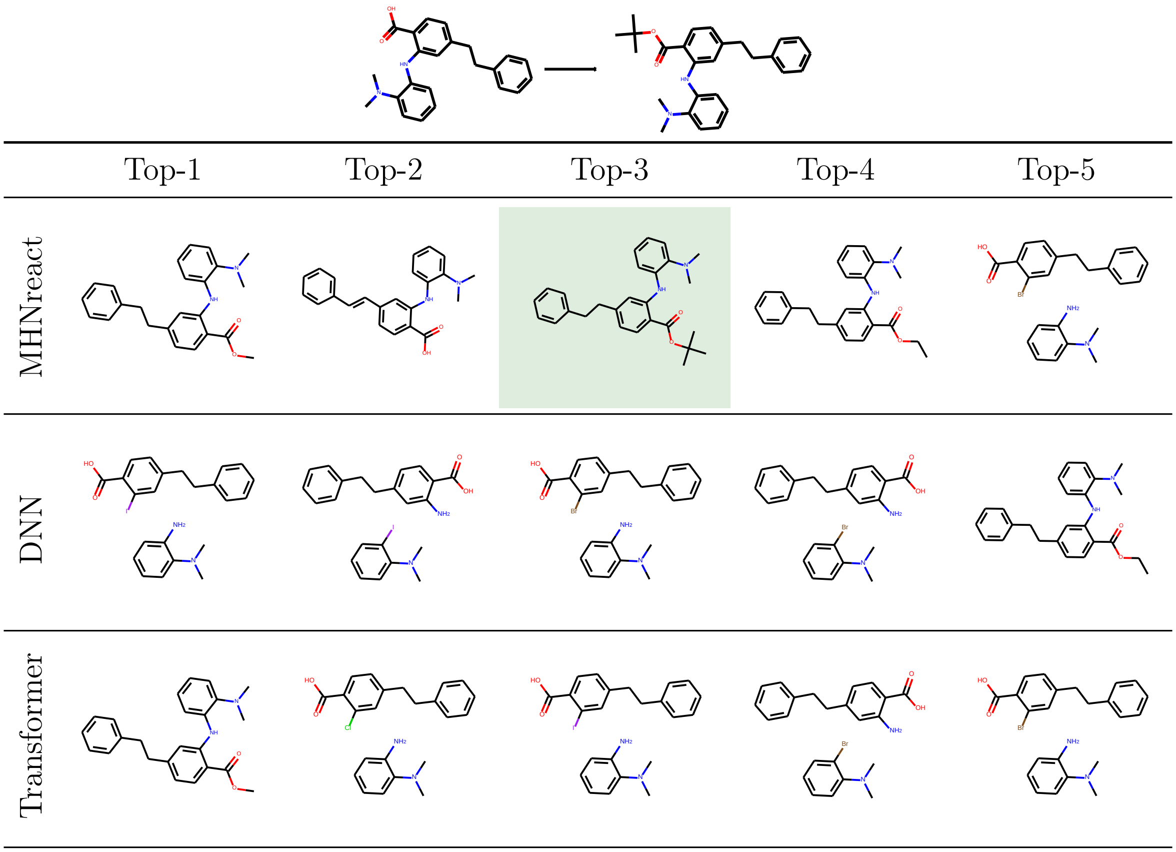

Figure S11: Sample #330 from the test-set.

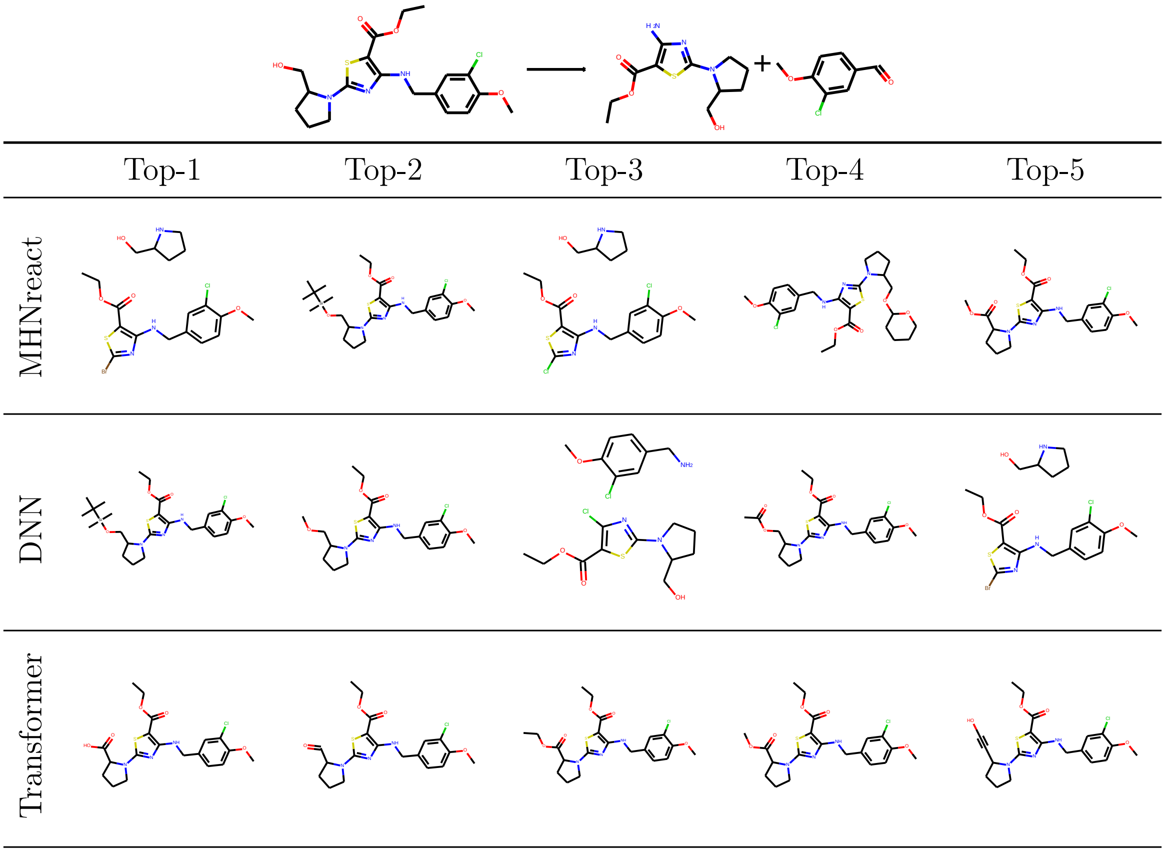

Figure S12: Sample #35 from the test-set. Ground-truth is not in one of the top-5 predictions.

S5 Hopfield Association Space

Figure S13 shows a t-Distributed Stochastic Neighbor Embedding (t-SNE) embedding of both the reaction fingerprints and the learned embeddings. Each point is colored according to its class as defined in[17].

For example, reactions belonging to the type `oxidations` can be distant in the fingerprint space (pink dots in the left plot in Figure S13), while in their learned representations are closer (pink dots in right figure). Note that our model did not have access to these reaction types. It can be seen that the chosen representation for reaction templates already captures information about the relationship, and the same reaction types are represented closer after embedding it using t-SNE.

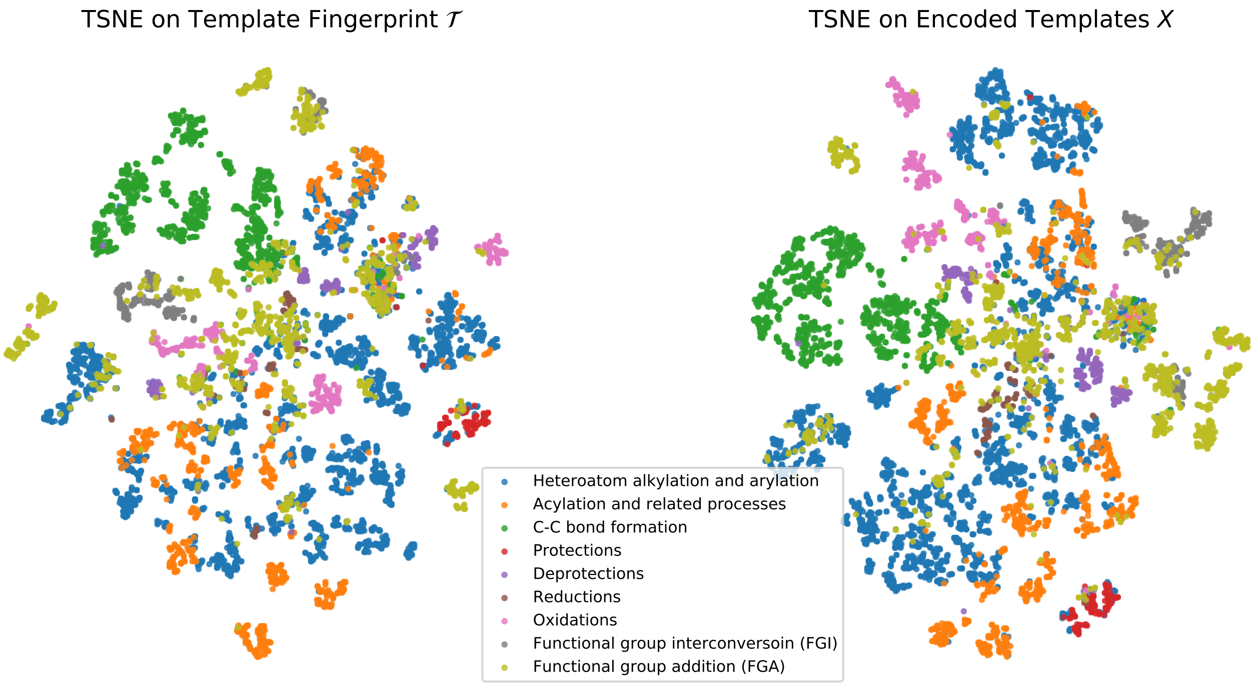

Figure S13: t-SNE downprojection of the reaction template fingerprints (left) and learned representations of reaction templates $\boldsymbol{X}$ (right). The colors represent reaction types of substructure-based expert systems as categorized by[17].

S6 Illustrative Example for Single-Step Retrosynthesis

We want to predict how to synthesize a given molecule as seen in Figure S14. First the molecule as well as multiple templates are encoded. Many templates could be applicable and would produce reactants, but we only want to predict relevant templates, those that are likely to correspondond to realistic reactions. MHNreact encodes the templates and associates them with the learned molecular representation and returns a ranking of the templates. Through encoding the templates, even templates with few training-data can be predicted. The templates are executed on the molecule in order of the provided ranking, in this case only the top-ranked template, which gives the reactants from which the desired molecule can likely be synthesized.

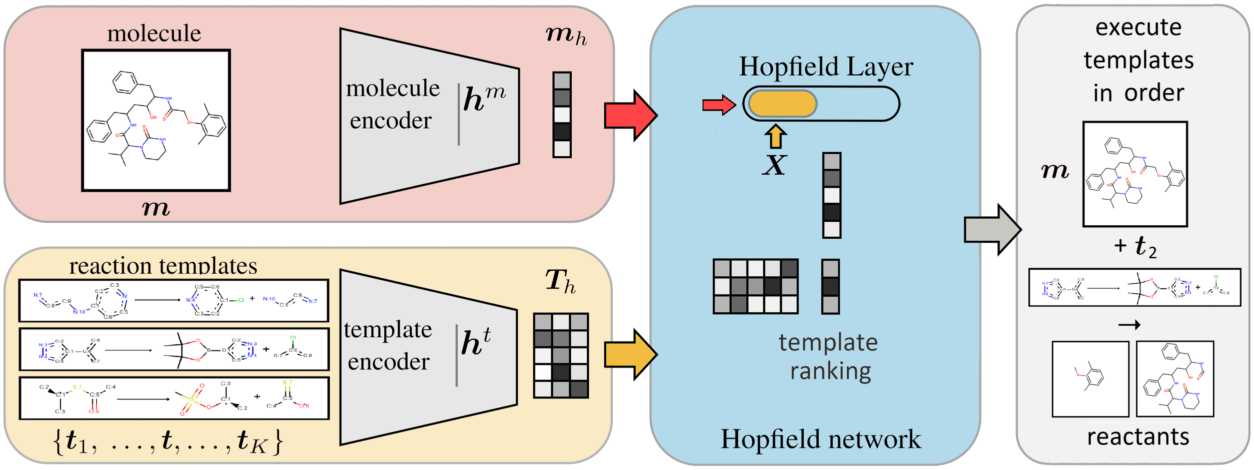

Figure S14: Illustration of the process for single-step retrosynthesis

S7 Objective and loss functions

Loss on retrieved patterns. We provide a more general view on the objective and the loss function from the perspective of Hopfield networks and retrieving patterns. The main idea is to retrieve patterns from label space, rather than from Hopfield space, because the loss functions operate in the label space. The last Hopfield layer of our architecture supplies both $\boldsymbol{p}$, the softmax vector of probabilities of drawing reaction templates, and $\boldsymbol{\xi}^{\text{new}}$, an average of reaction template representations $\boldsymbol{x}$. However, averages of reaction templates are no longer reaction templates, but we are interested in the *probability* ℓ_{ξ} of drawing an $\boldsymbol{x}_k$ that fits to $\boldsymbol{\xi}$.

The probability ℓ_ξ can still be computed via a slightly modified Hopfield network update, where instead of retrieving from a memory $\boldsymbol{X}$ of template representations in Hopfield space, we retrieve from the space of labels or scores. Such an update has been introduced previously and uses stored patterns that are augmented by labels[35] p.83ff.

The probability ℓ_ξ of drawing a $\boldsymbol{x}_k$ that fits to $\boldsymbol{\xi}$ can be computed by a modified Hopfield network update:

$$\boldsymbol{q}_\xi \ = \ \boldsymbol{L}\, \boldsymbol{p} \ = \ \boldsymbol{L}\, \mathrm{softmax}(\beta \boldsymbol{X}^T \boldsymbol{\xi}) \ , \quad \ell_\xi \ = \ \mathbf{1}^T \, \boldsymbol{q}_\xi \ , \tag{2}$$

where $\boldsymbol{L} \in \mathbb{R}^{K \times K}$ is the diagonal matrix of labels that are zero or one and $\mathbf{1} \in \mathbb{R}^K$ is the vector of ones. In general, $\boldsymbol{L}$ can be used to include unlabeled data points as stored patterns, where $\boldsymbol{L}$ is a Gram matrix times a diagonal label matrix to transfer label information from labeled data points to unlabeled data points. $L_{kk} = 1$ means that $\boldsymbol{x}_k$ fits to $\boldsymbol{\xi}$ and $L_{kk} = 0$ means that $\boldsymbol{x}_k$ does not fit to $\boldsymbol{\xi}$. In the context of the Hopfield networks, if more than one $\boldsymbol{x}_k$ fits to $\boldsymbol{\xi}$ then all $\boldsymbol{x}_k$ that fit to $\boldsymbol{\xi}$ constitute a metastable state. Instead of L_{kk} being equal to zero or one, L_{kk} can give a non-negative score for how well $\boldsymbol{x}_k$ does fit to $\boldsymbol{\xi}$. In this case ℓ_ξ is the *expected score.*

In this general view, our objective is to minimize $-\log(\ell_\xi)$. If L_{kk} is equal to zero or one, $\log(\ell_\xi)$ is the log-likelihood of drawing a fitting $\boldsymbol{x}_k$. If only one $\boldsymbol{x}_k$ fits (exactly one L_{kk} is one), then our objective is equivalent to the cross-entropy (CE) loss for multi-class classification. However, if more $\boldsymbol{x}_l$ are labeled as fitting, then our objective is different from CE, which might not be appropriate. If L_{kk} is a non-negative score for the molecule-template pair, our approach will maximize the expected score.

Cross-entropy loss. In a simple setting, in which each molecule only has a single correct reaction template in $\boldsymbol{T}$, a categorical cross-entropy loss is equivalent to the suggested loss. We encode the correct template by a one-hot vector $\boldsymbol{y} = (0, \ldots, 0, 1, 0, \ldots, 0)$, where 1 indicates the position of the correct template in the template set $\boldsymbol{t} = \boldsymbol{t}^k$. We then minimize the

cross-entropy loss function $\ell_{\mathrm{CE}}(\boldsymbol{y}, \boldsymbol{p}) = \mathrm{crossentropy}(\boldsymbol{y}, \boldsymbol{p})$ between ground truth $\boldsymbol{y}$ and the model's predictions $\boldsymbol{p}$ for a single pair of the training set and the overall loss is an average over all such pairs. The corresponding algorithm is given in Alg. 1.

Contrastive loss in Hopfield space. An alternative to the cross-entropy loss would be to use a contrastive loss on the retrieved pattern $\boldsymbol{\xi}^{\mathrm{new}}$. This contrastive loss measures the cosine similarity of the retrieved pattern with the correct stored patterns with the InfoNCE function[13,36]:

$$\ell_{\mathrm{p}}(\boldsymbol{\xi}^{\mathrm{new}}, \boldsymbol{x}^{+}, \boldsymbol{X}^{-}) = \mathrm{InfoNCE}(\boldsymbol{\xi}^{\mathrm{new}}, \boldsymbol{x}^{+}, \boldsymbol{X}^{-}) \tag{3}$$

where sim(., .) is a pairwise similarity function, $\boldsymbol{x}^{+}$ is the representation of the correct reaction templates, and $\boldsymbol{X}^{-}$ is the set of representations of the incorrect reaction templates, that are contrasted against each other. This loss is equivalent to cross-entropy loss if a) $1/\tau = \beta$, b) the similarity function is the dot product, and c) $\boldsymbol{\xi}$ is used instead of $\boldsymbol{\xi}^{\mathrm{new}}$. Our experiments show that this loss can lead to models with comparable performance to those trained with cross-entropy loss. The according algorithm with pattern loss as alternative loss is shown in Alg. 1. We envision that advances in estimating mutual information[37,38] and contrastive learning[39] could lead to improved zero- and few-shot capabilities of our model.

We provide a formulation of our method as simplified pseudo-code in a Python/Pytorch[12]-like language (see Algorithm 1). The pseudo-code provides a version with two stacked Hopfield layers and three possible formulations of the loss function.

Algorithm 1 MHN for reaction template prediction (simplified, e.g. skip-connections omitted).

```
#mol_encoder() — e.g. fully-connected or MPNN. Maps to dimension d_m.
#template_encoder1() Maps to dimension d_t1.
#template_encoder2() Maps to dimension d_t2.
#m_train, t_train — pair of product molecule and reaction template from training set
#T — set of K reaction templates including t_train
#d — dimension of Hopfield space

## FORWARD PASS
T1_h = template_encoder1(T)#[d_t1,K]
T2_h = template_encoder2(T)#[d_t2,K]
m_h = mol_encoder(m_train)#[d_m,1]
xinew1,_,_ = Hopfield(m_h,T1_h,dim=d)
xinew,p,X = Hopfield(xinew1,T2_h,dim=d)
p=pool(p,axis=1) #[K,1]

## LOSS
# cross-entropy loss, association loss
label = where(T==t_train)#[K,1]
loss = cross_entropy(p,label)
# alternative 1:  Hopfield loss
L = diag(where(T==t_train))#[K,K]
loss = -log(sum(L@p))
# alternative 2:  contrastive loss
label = where(T==t_train)#[K,1]
pos = X[label] #[d,1]
neg_label = where(T!=t_train)#[K,1]
neg = X[neg_label] #[d,K-1]
loss = -InfoNCE(xinew,pos,neg)
```

S8 List of Acronyms

CASP computer-assisted synthesis planning

CLIP Contrastive Language–Image Pre-training

ConVIRT Contrastive VIsual Representation Learning from Text

DNN deep neural network

ECFP extended connectivity fingerprint

FPF fingerprint filter

FP fingerprint

GLN graph logic network

InfoNCE Information Noise-Contrastive Estimation

MACCS molecular access system

MHFP mini-hash fingerprint

MHN modern Hopfield network

ML machine learning

NN neural network

SELU self-normalizing linear unit

SMARTS SMILES arbitrary target specification

SMILES simplified molecular-input line-entry system

USPTO United States patent and trademark office

t-SNE t-Distributed Stochastic Neighbor Embedding

References

(1) Baylon, J. L.; Cilfone, N. A.; Gulcher, J. R.; Chittenden, T. W. Enhancing Retrosynthetic Reaction Prediction with Deep Learning Using Multiscale Reaction Classification. *J. Chem. Inf. Model.* **2019**,

(2) Ishida, S.; Terayama, K.; Kojima, R.; Takasu, K.; Okuno, Y. Prediction and Interpretable Visualization of Retrosynthetic Reactions Using Graph Convolutional Networks. *J. Chem. Inf. Model.* **2019**, *59*, 5026–5033.

(3) Fortunato, M. E.; Coley, C. W.; Barnes, B. C.; Jensen, K. F. Data Augmentation and Pretraining for Template-Based Retrosynthetic Prediction in Computer-Aided Synthesis Planning. *J. Chem. Inf. Model.* **2020**, *60*, 3398–3407.

(4) Segler, M. H. S.; Waller, M. P. Neural-Symbolic Machine Learning for Retrosynthesis and Reaction Prediction. *Chemistry* **2017**, *23*, 5966–5971.

(5) Segler, M. H. S.; Preuss, M.; Waller, M. P. Planning Chemical Syntheses with Deep Neural Networks and Symbolic AI. *Nature* **2018**, *555*, 604–610.

(6) Bjerrum, E. J.; Thakkar, A.; Engkvist, O. Artificial Applicability Labels for Improving Policies in Retrosynthesis Prediction. *ChemRxiv* **2020**,

(7) Dai, H.; Li, C.; Coley, C.; Dai, B.; Song, L. Retrosynthesis Prediction with Conditional Graph Logic Network. *Adv. Neural Inf. Process. Syst.* **2019**, *32*, 8872–8882.

(8) Sun, R.; Dai, H.; Li, L.; Kearnes, S.; Dai, B. Energy-based View of Retrosynthesis. *arXiv (Machine Learning)* **2020**,

(9) Coley, C. W.; Green, W. H.; Jensen, K. F. RDChiral: An RDKit Wrapper for Handling Stereochemistry in Retrosynthetic Template Extraction and Application. *J. Chem. Inf. Model.* **2019**, *59*, 2529–2537.

(10) Morgan, H. L. The Generation of a Unique Machine Description for Chemical Structures-A Technique Developed at Chemical Abstracts Service. *J. Chem. Doc.* **1965**, *5*, 107–113.

(11) Landrum, G. RDKit: Open-Source Cheminformatics. **2006**, accessed on 2020-01-01.

(12) Paszke, A.; Gross, S.; Massa, F.; Lerer, A.; Bradbury, J.; Chanan, G.; Killeen, T.; Lin, Z.; Gimelshein, N.; Antiga, L.; Desmaison, A.; Köpf, A.; Yang, E.; DeVito, Z.; Raison, M. Tejani, A.; Chilamkurthy, S.; Steiner, B.; Fang, L.; Bai, J.; Chintala, S. Pytorch: An imperative style, high-performance deep learning library. *Adv. Neural Inf. Process. Syst.* **2019**, *32*, 8026–8037.

(13) Oord, A. v. d.; Li, Y.; Vinyals, O. Representation learning with contrastive predictive coding. *arXiv (Machine Learning)* **2018**,

(14) Ba, J. L.; Kiros, J. R.; Hinton, G. E. Layer normalization. *arXiv (Machine Learning)* **2016**,

(15) Loshchilov, I.; Hutter, F. Decoupled weight decay regularization. *arXiv (Machine Learning)* **2017**,

(16) Coley, C. W.; Rogers, L.; Green, W. H.; Jensen, K. F. Computer-Assisted Retrosynthesis Based on Molecular Similarity. *ACS Cent. Sci.* **2017**, *3*, 1237–1245.

(17) Schneider, N.; Lowe, D. M.; Sayle, R. A.; Landrum, G. A. Development of a Novel Fingerprint for Chemical Reactions and Its Application to Large-Scale Reaction Classification and Similarity. *J. Chem. Inf. Model.* **2015**, *55*, 39–53.

(18) Tetko, I. V.; Karpov, P.; Van Deursen, R.; Godin, G. State-of-the-Art Augmented NLP Transformer Models for Direct and Single-Step Retrosynthesis. *Nat. Commun.* **2020**, *11*, 5575.

(19) Probst, D.; Reymond, J.-L. A probabilistic molecular fingerprint for big data settings. *J. Cheminf.* **2018**, *10*, 1–12.

(20) Klambauer, G.; Unterthiner, T.; Mayr, A.; Hochreiter, S. Self-normalizing neural networks. *Adv. Neural Inf. Process. Syst.* **2017**, 972–981.

(21) Rogers, D.; Hahn, M. Extended-Connectivity Fingerprints. *J. Chem. Inf. Model.* **2010**, *50*, 742–754.

(22) Yan, C.; Ding, Q.; Zhao, P.; Zheng, S.; Yang, J.; Yu, Y.; Huang, J. RetroXpert: Decompose Retrosynthesis Prediction like a Chemist. *arXiv (Machine Learning)* **2020**,

(23) Somnath, V. R.; Bunne, C.; Coley, C. W.; Krause, A.; Barzilay, R. Learning graph models for template-free retrosynthesis. *arXiv (Machine Learning)* **2020**,

(24) Lee, H.; Ahn, S.; Seo, S.-W.; Song, Y. Y.; Hwang, S.-J.; Yang, E.; Shin, J. RetCL: A Selection-Based Approach for Retrosynthesis via Contrastive Learning. *arXiv (Machine Learning)* **2021**,

(25) Hasic, H.; Ishida, T. Single-Step Retrosynthesis Prediction Based on the Identification of Potential Disconnection Sites Using Molecular Substructure Fingerprints. *J. Chem. Inf. Model.* **2021**, *61*, 641–652.

(26) Guo, Z.; Wu, S.; Ohno, M.; Yoshida, R. A Bayesian Algorithm for Retrosynthesis. *J. Chem. Inf. Model.* **2020**, *60*, 4474–4486.

(27) Schwaller, P.; Laino, T.; Gaudin, T.; Bolgar, P.; Hunter, C. A.; Bekas, C.; Lee, A. A. Molecular Transformer: A Model for Uncertainty-Calibrated Chemical Reaction Prediction. *ACS Cent. Sci.* **2019**, *5*, 1572–1583.

(28) Ishiguro, K.; Ujihara, K.; Sawada, R.; Akita, H.; Kotera, M. Data Transfer Approaches to Improve Seq-to-Seq Retrosynthesis. *arXiv (Machine Learning)* **2020**,

(29) Ucak, U. V.; Kang, T.; Ko, J.; Lee, J. Substructure-based neural machine translation for retrosynthetic prediction. *J. Cheminf.* **2021**, *13*, 4.

(30) Liu, B.; Ramsundar, B.; Kawthekar, P.; Shi, J.; Gomes, J.; Luu Nguyen, Q.; Ho, S.; Sloane, J.; Wender, P.; Pande, V. Retrosynthetic Reaction Prediction Using Neural Sequence-to-Sequence Models. *ACS Cent. Sci.* **2017**, *3*, 1103–1113.

(31) Karpov, P.; Godin, G.; Tetko, I. V. A transformer model for retrosynthesis. *Int. Conf. on Artif. Neur. Netw.* **2019**, 817–830.

(32) Wang, X.; Li, Y.; Qiu, J.; Chen, G.; Liu, H.; Liao, B.; Hsieh, C.-Y.; Yao, X. RetroPrime: A Diverse, Plausible and Transformer-Based Method for Single-Step Retrosynthesis Predictions. *Chem. Eng. J.* **2021**, *420*, 129845.

(33) Sacha, M.; Błaz, M.; Byrski, P.; Dabrowski-Tumansski, P.; Chrominsski, M.; Loska, R.; Włodarczyk-Pruszynski, P.; Jastrzebski, S. Molecule edit graph attention network: modeling chemical reactions as sequences of graph edits. *J. Chem. Inf. Model.* **2021**, *61*, 3273–3284.

(34) Lowe, D. M. Extraction of chemical structures and reactions from the literature. Ph.D. thesis, University of Cambridge, 2012.

(35) Ramsauer, H.; Schäfl, B.; Lehner, J.; Seidl, P.; Widrich, M.; Gruber, L.; Holzleitner, M.; Adler, T.; Kreil, D.; Kopp, M. K.; Klambauer, G.; Brandstetter, J.; Hochreiter, S. Hopfield Networks is All You Need. *Int. Conf. Learn. Rep.* **2021**,

(36) Chen, T.; Kornblith, S.; Norouzi, M.; Hinton, G. A simple framework for contrastive learning of visual representations. *Int. Conf. Mach. Learn.* **2020**, 1597–1607.

(37) Poole, B.; Ozair, S.; vanDenOord, A.; Alemi, A. A.; Tucker, G. On Variational Bounds of Mutual Information. *Proc. 36th Int. Conf. Mach. Learn.* **2019**, *97*, 5171–5180.

(38) Cheng, P.; Hao, W.; Dai, S.; Liu, J.; Gan, Z.; Carin, L. CLUB: A Contrastive Log-ratio Upper Bound of Mutual Information. *Proc. 37th Int. Conf. Mach. Learn.* **2020**, *119*, 1779–1788.

(39) Fürst, A.; Rumetshofer, E.; Tran, V.; Ramsauer, H.; Tang, F.; Lehner, J.; Kreil, D.; Kopp, M.; Klambauer, G.; Bitto-Nemling, A.; Hochreiter, S. CLOOB: Modern Hopfield Networks with InfoLOOB Outperform CLIP. *arXiv (Machine Learning)* **2021**,
